# Microclimate variability impacts the coexistence of highland and lowland ectotherms

**DOI:** 10.1111/1365-2656.70030

**Published:** 2025-03-20

**Authors:** Urban Dajčman, Urtzi Enriquez‐Urzelai, Anamarija Žagar

**Affiliations:** ^1^ Biotechnical Faculty University of Ljubljana Ljubljana Slovenia; ^2^ Department of Organisms and Ecosystems Research National Institute of Biology Ljubljana Slovenia; ^3^ Institute of Vertebrate Biology Czech Academy of Sciences Brno Czech Republic; ^4^ CIBIO, Centro de Investigação em Biodiversidade e Recursos Genéticos, InBIO Laboratório Associado Universidade do Porto Vairão Portugal

**Keywords:** dynamic energy budget, ectotherms, elevation, Lacertidae, life history, microclimate, syntopy

## Abstract

1. Understanding differences in life‐history outcomes under variable abiotic conditions is essential for understanding species coexistence. At middle elevations, a mosaic of available sets of abiotic conditions could allow highland and lowland species of the same ecological guild to overlap. Therefore, these sites are excellent to study the influence of abiotic conditions on life history and, thus, spatial overlap patterns of competing species.
2. To test differences in life‐history outcomes, we selected a pair of closely related lacertids, *Iberolacerta horvathi* and *Podarcis muralis*, with an overlapping geographical range but a contrasting elevational distribution. To assess how abiotic and biotic factors contribute to the realized niches of both species, we first built dynamic energy budget (DEB) models for each species based on available functional and life‐history data. Then, we used a mechanistic modelling framework (NicheMapR) to simulate the microclimatic conditions at 15 study sites across an elevational gradient and performed whole life‐cycle simulations for both species to compare egg development times, lifespans, reproductive years, mean yearly basking and foraging times and yearly fecundity in syntopy and allotopy along the elevational gradient.
3. Our simulations show that the variability of abiotic conditions along an elevational gradient affects life‐history traits of both species. We found strong effects of species and elevation on life‐history outcomes such as longevity, activity and fecundity. We also observed the effects of syntopy/allotopy on egg development times, activity and reproductive output. In addition, we found a significant interplay between elevation and species impacting fecundity where occupying higher elevation habitats resulted in a more pronounced reduction in fecundity in *P. muralis*. Furthermore, using two different thermal preferences for spring and summer, we show that some physiological and reproductive traits change with seasonal changes in thermal preferences.
4. Based on our simulations, we conclude that the intermediate elevations that harbour the majority of syntopic populations exhibit high environmental variability that is likely facilitating species coexistence. Since our model predictions support that the current elevational distribution of the species is not only affected by abiotic factors, this suggests that past historical contingencies might have also played a significant role.
5. Our study provides a framework using mechanistic models to understand current distribution patterns of two interacting species by comparing life‐history differences between species based on responses to changing abiotic conditions along an elevation gradient.

## Graphical abstract

Our paper is an original attempt to use mechanistic models to explain the patterns of species coexistence along an environmental gradient. It is novel both in its methodological approach to the topic as well as through the obtained results.

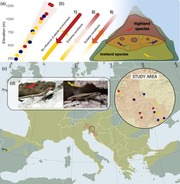

## 1 INTRODUCTION

At the local scale, biotic interactions, such as interspecific competition, play a major role in determining the pool of species within communities (i.e. Eltonian niche; Elton, 1927), which can be alleviated through niche segregation or resource partitioning (Arrizabalaga‐Escudero et al., 2018; Reif et al., 2018). Therefore, species coexistence has long attracted the attention of ecologists (Gause, 1934; Grinnell, 1917; MacArthur, 1958; Pianka, 1973) and remains a vibrant field of research (Buckley & Roughgarden, 2006; Gravel et al., 2011; Tokeshi, 2009). Moreover, abiotic factors (e.g. climate and elevation) as well as dispersal filters (e.g. geographic barriers) all affect the coexistence of ecologically similar species (Brown & Carnaval, 2019). Ecologically similar species can have partially overlapping ranges with syntopic populations, where they coexist, and competition is possible (MacArthur, 1984; Pollock et al., 2014). Examples of such partly overlapping ranges are often observed due to transitions in climatic gradients such as elevational gradients where species often coexist in middle elevations (Colwell & Lees, 2000; McCain, 2005) or at specific latitudes (Reif et al., 2018).

Several alternative hypotheses can explain the co‐occurrence of ecologically similar species at intermediate levels of environmental gradients such as elevation. First, the co‐occurrence of species at middle elevations may be due to the biogeographic history of species (Monasterio et al., 2011). In this case, abiotic factors do not necessarily define the overlapping ranges, since the current distribution of species is influenced by past species' distributions along with their dispersal abilities (Guisan & Thuiller, 2005), with or without past niche differentiation in geographically isolated areas (Tang & Zhou, 2011). This follows the neutral theory of community ecology (Chesson, 2000; Hubbell, 2001) according to which species that are members of the same ecological guild have no significant ecological differences, and their distribution is a matter of ecological drift. The main hypothesis within neutral theory is functional equivalence; trophically similar species are demographically identical on a per capita basis in terms of birth, death and dispersal (Hubbell, 2001). Second, middle elevations may represent extreme conditions for both species, which are otherwise adapted to either lowland or highland conditions (Herzog et al., 2005). In such cases, species coexist at middle elevations because the abiotic conditions are less favourable there and competition between species is weakened (i.e. the stress gradient hypothesis; Bertness & Callaway, 1994; Maestre et al., 2009). A third possibility is that species at intermediate elevations diverge in resource utilization due to character displacement after secondary contact that facilitates their coexistence (Brodie et al., 2018; Chesson, 2000). In this final example, middle elevations provide an abundance of abiotic resources leading to possible competitive release (Begon & Townsend, 2021) enabling both species to realize their ecological niches and coexist.

Ectothermic animals, such as lizards, are largely influenced by abiotic factors, especially temperature (Giacometti et al., 2024), which changes with elevation. The body temperature of lizards is the most important physiological trait reflecting their performance (Huey & Pianka, 1981) and it depends on factors as diverse as common ancestry, geography, climate and behaviour (Giacometti et al., 2024; Grigg & Buckley, 2013). An example of three *Liopholis* lizards replacing each other along an elevational gradient between sea level and up to 2170 m above sea level showed that intraspecific variation in the thermal traits of lizards, rather than differences in thermal tolerances, maintains the distribution of species (Senior et al., 2019). It has also been shown that the phenotypic plasticity of basking behaviour differs between populations and species of *Niveoscincus* lizards between sea level and mountain top localities higher than 1100 m above sea level (Caldwell et al., 2017). Species and populations in the highlands were basking more than those in the lowlands (Caldwell et al., 2017). Thus, as thermoregulatory behaviour and body temperatures of lizards can change seasonally, ontogenetically and across environmental gradients in response to abiotic and biotic factors, mechanistic models of population growth that incorporate spatio‐temporal dynamics (Angilletta Jr et al., 2019) and thermodynamic aspects of the fundamental niche at the individual level (Kearney, 2019) are an excellent opportunity to study lizard coexistence.

Mechanistic modelling provides a contemporary approach to studying the ecological mechanisms underlying the patterns of spatial segregation of species (e.g. Briscoe et al., 2019; Gravel & Massol, 2020; Marn et al., 2022). From a thermodynamic perspective, the fundamental niche of an organism in a mechanistic niche model is characterized by constraints on its heat, mass (e.g. hydration state) and nutrient budgets in relation to growth, development and reproduction (Kearney & Porter, 2009). This is based on the premise that the set of environmental conditions that allow homeostasis during the life cycle of a given organism represents its fundamental niche (Kearney, 2012). As already mentioned before, for ectotherms, such as lizards, a critically important factor impacting their life histories is their thermal environment (Adolph & Porter, 1996) and many previous studies have demonstrated the importance of the thermal environment for various performance and fitness‐related traits (e.g. Adolph & Porter, 1996; Sears & Angilletta, 2004; Zamora‐Camacho et al., 2013). Apart from temperature, previous studies have also demonstrated the importance of other elevation‐dependent abiotic factors such as precipitation and light intensity (Santillán et al., 2018; Watkins Jr. et al., 2006), which may also affect lizard performance (Anderson et al., 2022; Clusella‐Trullas et al., 2011). These types of abiotic factors can be accounted for in mechanistic models (Meyer et al., 2023).

In our study, we rely on dynamic energy budget theory (DEB) (Kooijman, 2009; Marques et al., 2018), which is a mathematically formalized metabolic theory explaining the complete energy and mass balances from individuals to higher levels of biological organization (Kooijman, 2009; Nisbet et al., 2000). As such, it provides a framework to model energy and nutrient uptake and use throughout an organism's life cycle from first principles; namely, it is based on established physical laws (Kooijman, 2009; Nisbet et al., 2000; van der Meer, 2006). In addition to DEB theory, the principles of biophysical ecology provide complementary theory and equations to infer heat and water exchange rates between organisms and their environment (Briscoe et al., 2023). The combination of both approaches (i.e. DEB and biophysical ecology) allows us to simulate the growth, development and reproduction of organisms in their environment (Kearney & Porter, 2019). A recent example observing abiotic constraints on lizard reproduction, combining approaches from DEB theory and biophysical ecology, indicated that environmental constraints (temperature and season length) explain the observed pattern of litter frequency (Schwarzkopf et al., 2016). By predicting how life‐history traits and reproductive performance vary across elevation, we can gain insights into the interaction between microclimatic conditions along environmental gradients and the functional traits of species, and consequently better understand how microclimates and traits affect fitness and spatial segregation in pairs of elevationally segregated species. The described modelling approach is a valuable tool that complements empirical work observing the relationship between microhabitat and functional traits, especially in the context of energy balance (metabolic rates, water loss, energy allocation, Giacometti et al., 2022; Meter et al., 2020; Plasman et al., 2020; Tieleman et al., 2003).

**FIGURE 1:**
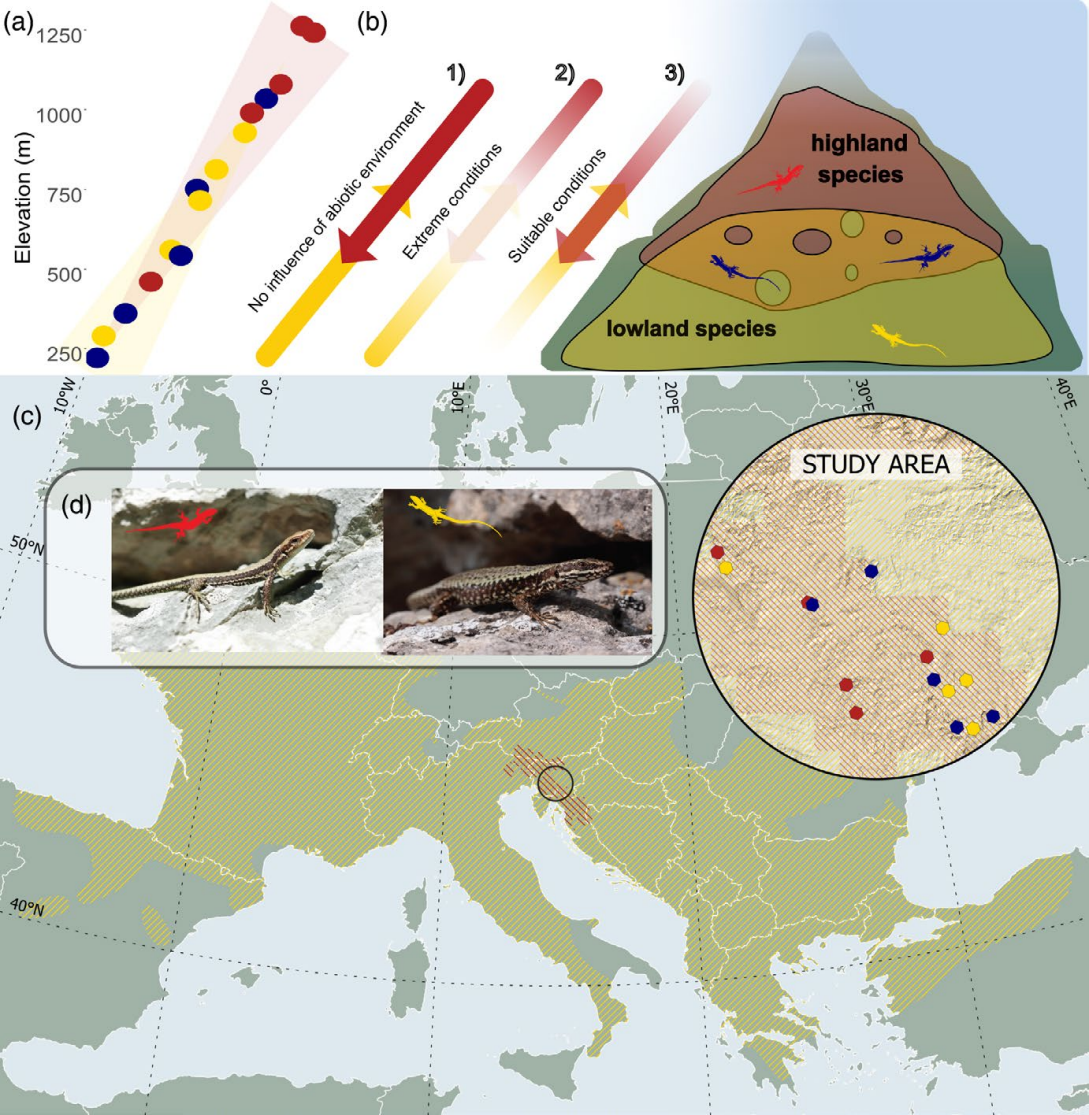
*Iberolacerta horvathi* (denoted in red) and *Podarcis muralis* (denoted in yellow) were studied across an elevation range between 250 and 1250 m above sea level and across allotopic and syntopic sites (syntopy—denoted in blue) (a) distribution of study sites across elevation with relative densities represented with the shaded triangles based on previous data collected in the study site (Žagar, 2016). (b) Main hypothesis: (1) the current distribution is a matter of historical and dispersal factors, and range overlap is not explained by the environment, (2) middle elevations represent the least optimal conditions for both species, thus representing a shared area of unfavourable abiotic conditions, (3) middle elevations provide favourable and resource abundant microclimatic conditions allowing both species to sustain viable populations. (c) Map of the study area with study sites. (d) Study species (Photo credits: Miha Krofel).

Here, we developed biophysical and DEB models for two ecologically similar lizard species, *Podarcis muralis* and *Iberolacerta horvathi*, with partial elevational segregation (Sillero et al., 2014). Specifically, we modelled the microclimatic conditions and functional life‐history traits (thermoregulatory behaviour, growth, activity, reproduction and lifespan) in known sites and populations where only one (allotopy) or both species (syntopy) occur along an elevational gradient (Figure 1a) (Žagar, 2016). To test the influence of environmental factors on the coexistence patterns at middle elevations, we defined three hypotheses (Figure 1b). The first is a neutral theory of community ecology‐based hypothesis (Hubbell, 2001) and predicts that the current distribution is a matter of historical and dispersal factors, and range overlap (i.e. syntopy) is not explained by the environment. Predictions showing the same response of the two species to both locations and elevation in life‐history traits would suggest such a situation. Second, we consider the stress gradient hypothesis, which states that middle elevations represent the suboptimal conditions for both species, thus representing a shared area of unfavourable abiotic conditions (Bertness & Callaway, 1994; Chesson & Huntly, 1997). The third hypothesis is the abundance hypothesis constructed around the idea that middle elevations provide favourable and resource abundant microclimatic conditions allowing both species to sustain viable populations (Brodie et al., 2018; Chesson, 2000). By comparing the possible outcomes, we try to interpret the importance of underlying abiotic factors for the functional diversity in ecosystems.

## 2 MATERIALS AND METHODS

### 2.1 Data collection on life‐history traits

To ensure the validity of our models, we collected data from available historical and contemporary sources on morphology, behaviour, physiology and life‐history traits such as egg development times, clutch sizes, preferred body temperature and critical thermal minima and maxima for our model species (Table S1). Additionally, we included data on environmental temperatures (*T*_e_) of active lizards in the study area obtained from previous field surveys (Žagar et al., 2023). These data were used for the DEB and the biophysical modelling approach explained below. Our model species are *Iberolacerta horvathi* and *Podarcis muralis*, a pair of lacertid lizards that are partially elevationally segregated throughout their sympatric range in the south‐eastern Alps and northern Dinaric Mountains (Sillero et al., 2014). Both species are morphologically similar in the study area (Figure 1), with small differences in weight and head height, which are all higher in *P. muralis* (Žagar et al., 2017). *Podarcis muralis* also has a stronger bite force than *I. horvathi*; therefore, it might be able to catch larger prey, as well as have a competitive advantage in interspecific agonistic interactions that are common between male lizards (Žagar et al., 2017). Both species are oviparous (Speybroeck et al., 2016) and lay up to five eggs per clutch, although *P. muralis* has a higher fecundity, often having several (up to three) clutches in a single year (De Luca, 1989; Ji & Brana, 2000). Although there are reports of high clutch sizes in *P. muralis* (up to 11 eggs; Ji & Brana, 2000), we chose to limit clutches to a maximum of five eggs in our models for *P. muralis*, based on previous reports from a geographically closer region (Sacchi et al., 2012). *Podarcis muralis* shows a faster pace of life than *I. horvathi*. While *P. muralis* becomes sexually mature at the age of about 2 years and lives up to 10 years (Castanet, 1994; Castanet & Roche, 1981), *Iberolacerta horvathi* becomes sexually mature at three and lives up to 9 years (Sindaco et al., 2006). Previous studies conducted in our study area show that both species have similar mean preferred body temperatures in summer but differ in *T*_pref_ in spring (Osojnik et al., 2013). Both species have similar standard metabolic rates, but the measurement of the maximum capacity of metabolism at the subcellular level, measured as the iodonitrotetrazolium (INT) reduction capacity, showed that *I. horvathi* has a higher metabolic potential activity (Žagar, Simčič, et al., 2015). Additionally, water loss rates were higher in *P. muralis* (Osojnik et al., 2013). Past studies from the region also revealed that our study system exhibits evidence of interference competition (e.g. Žagar, Carretero, et al., 2015) and potential indirect competition (via shared predators or parasites, e.g. Dajčman et al., 2022; Žagar, Bitenc, et al., 2015) among current contact zones.

### 2.2 Species distribution and study locations

*Podarcis muralis* and *Iberolacerta horvathi* may occur in syntopy or allotopy in different areas where the species ranges overlap (Krofel et al., 2009; Rassati, 2010; Žagar, 2008; Žagar et al., 2007). The abundance of *I. horvathi* is higher at high altitudes, while *P. muralis* is denser at lower ones. Our previous work has shown that, in our study area, *I. horvathi* had the highest relative abundances between 900 and 1099 m above sea level, while *P. muralis* had the highest abundances between 100 and 499 m above sea level (Žagar, 2016). Syntopic populations occur throughout the study area and at all altitudes, with most syntopic populations found at middle elevations (Cabela et al., 2007; Lapini et al., 2004; Rassati, 2010; Žagar, 2016; Figure 1), averaging 620 m above sea level. Based on these previous reports for our study region, we categorized *P. muralis* as ‘lowland’ species and *I. horvathi* as ‘highland’ species (Figure 1).

Our study area is in southern Slovenia (Figure 1c—Study area) where both species typically occupy open forest patches often characterized by rock cliffs or boulders, rocky outcrops or human‐made rock or stone structures such as sides of the roads and buildings (Žagar et al., 2013). We conducted field surveys between May and September 2022 to select 15 sites of occurrence: five syntopic, five allotopic *I. horvathi* and five allotopic *P. muralis*, covering the whole elevation range of occurrence of both species within the region (Table 1; Figure 1a). By conducting the field surveys, we assured that the locations used in our microclimatic models covered actual sites of known coexistence. All fieldwork was carried out in the scope of the permit 35601‐10/2021‐5 issued by the Slovene Environment Agency.

**TABLE 1:** Study site locations arranged by increasing elevation with information given on the occurrence of either both or only one species (syntopy or allotropy, respectively).

Location	Lat	Long	Elevation (m a.s.l.)	Species	Occurrence
Bilpa	45.513	14.962	238	Both	Syntopy
Sv. Štefan	45.480	14.886	307	*Podarcis muralis*	Allotopy
Iški vintgar	45.907	14.494	378	Both	Syntopy
Planinska jama	45.822	14.247	477	*Iberolacerta horvathi*	Allotopy
Kočevska Reka	45.582	14.791	578	*P. muralis*	Allotopy
Unška koliševka	45.816	14.265	578	Both	Syntopy
Ribnica	45.753	14.770	733	*P. muralis*	Allotopy
Kuželjska stena	45.484	14.823	769	Both	Syntopy
Kovk	45.915	13.928	830	*P. muralis*	Allotopy
Fridrihštajn	45.611	14.860	946	*P. muralis*	Allotopy
Velike bele stene	45.675	14.708	1007	*I. horvathi*	Allotopy
Kameni zid	45.613	14.735	1052	Both	Syntopy
Trnovski gozd	45.957	13.895	1097	*I. horvathi*	Allotopy
Snežnik	45.599	14.397	1259	*I. horvathi*	Allotopy
Orlovica	45.523	14.436	1279	*I. horvathi*	Allotopy

### 2.3 Biophysical and DEB model integration

We used the microclimate model of the R package NicheMapR v 3.3.2 (Kearney & Porter, 2017) to obtain microclimatic data for all 15 study locations (Figure 1; Table 1). NicheMapR's microclimate model is a set of open‐source routines to simulate the specific microclimates available to organisms at relevant temporal and spatial scales (Kearney & Porter, 2017). The simulations are based on terrain (e.g. soil properties, slope, aspect, elevation, available shade) and climate conditions (i.e. minimum, maximum temperatures, precipitation, cloud cover, humidity, wind speed and solar radiation). We used the *micro_ncep* function from NicheMapR, which uses the microclima package (Maclean et al., 2019), as well as the RNCEP (Kemp et al., 2012) and elevatr (Hollister et al., 2017) packages. These packages access 6‐hourly, 2.5 × 2.5° gridded weather data from NCEP (National Centers for Environmental Prediction) (Kalnay et al., 1996) and downscale it for terrain‐specific analysis, allowing us to model local climate conditions. Furthermore, *micro_ncep* downscales macroclimatic data accounting for local terrain effects (~30 × 30 m), elevation‐induced lapse rates, coastal influences and cold‐air drainage (Kearney, Gillingham et al., 2019). Soil properties in the chosen locations were obtained by accessing the SoilGrids database (a database of soil properties across the globe; Hengl et al., 2017), through the *micro_ncep* function. The microclimatic conditions were simulated for a range of 16 years from January 1995 to December 2010 to account for the maximum extreme known lifespan of species (Eroğlu et al., 2018), with the moisture and snow subroutines turned on. The period between 1995 and 2010 was chosen because most of the occurrence data of both species in the study sites was collected during that period (Žagar, 2016). The simulations for microclimatic conditions were run for all 15 locations. From the output, we selected the predictions on mean environmental temperature, number of days without snow cover, mean solar radiation and mean relative humidity for further statistical comparisons of microclimatic conditions between localities (see Section 2.4).

We performed the DEB parameter estimation for both species separately, using the ‘covariation method’. The ‘covariation method’ for estimating the parameters of the standard DEB model is explained in detail in Lika et al. (2011) and Marques et al. (2018). We used the standard DEB model for both lizard species. We performed the parameter estimation based on a combination of previously published and our own unpublished data (Tables S1 and S2), including information on age, size and weight at different life stages, maximum reproduction rate, mass‐length relationships, fecundity and metabolic potential for both species to obtain core DEB parameters (Table S3). In addition, the dataset for *P. muralis* included growth and temperature‐dependent development time. Parameters were estimated separately for each species using MATLAB (The MathWorks Inc., 2022) and the freely available AmPtool (AmPtool, 2022) and DEBtool_M (DEBtool, 2022) MATLAB packages. We checked for the goodness‐of‐fit of models with the estimated parameters via the mean relative error (MRE) and symmetric mean squared error (SMSE) statistics (Marques et al., 2018). The models and all parameter values with accompanying MATLAB scripts used for parameter estimation are available from the publicly accessible add‐my‐pet database (Marques et al., 2018; https://www.bio.vu.nl/thb/deb/deblab/add_my_pet/species_list.html). Detailed parameters of the model are presented in Tables S1 and S2, while mass and energy flows with a general description of the standard DEB model are presented in a schematic representation (Figure S1). DEB models for both species performed well at describing the life‐history and life cycle traits for the studied species, and predictions matched the data used for model parametrization (Table S3; Figure S2).

To simulate lizards' growth, development and reproduction in the study sites given the estimated microclimates, we used the *ectotherm* function of NicheMapR (Kearney & Porter, 2019). The ectotherm model first integrates output from the microclimate model with species functional traits (i.e. morphology, physiology and behaviour) to estimate variables such as body temperatures (*T*_b_) through the biophysical submodel, accounting for the processes of conduction, convection, radiation, evaporation and metabolic heat production (Kearney & Porter, 2019). Furthermore, based on body temperatures and DEB model parameters, it estimates changes in DEB state variables, thus allowing to simulate growth, development and reproductive output (Kearney & Porter, 2019). We ran simulations for both species at each of the 15 sites—regardless of whether they naturally co‐occur—so that *P. muralis* was also modelled at sites occupied only by *I. horvathi* and vice versa. The simulations started on the 140th day of the year, the time at which eggs are usually laid (Arribas & Galán, 2005; Ji & Brana, 2000). The egg was simulated to be buried 5 cm below ground for both species to account for oviposition in soil and rock cracks (Speybroeck et al., 2016). After hatching (i.e. for juveniles and adults), the animals were simulated to leave retreats when reaching minimum body temperatures necessary for activity to occur (*T*_rb_ = 17.8°C and 15.6°C for *P. muralis* and *I. horvathi*, respectively) obtained from personal unpublished data (Table S2). When active above‐ground, lizards were allowed to seek shade for thermoregulation (between 0% and 90% shade). We only allowed diurnal activity bounded between the voluntary thermal minima (spring: *T*_forage_min_ = 18.2 and 19.1.7°C; summer: *T*_forage_min_ = 26.3 and 22.2°C for *P. muralis* and *I. horvathi*, respectively) and maxima (spring: *T*_forage_max_ = 32.8 and 31.6°C; summer: *T*_forage_max_ = 35.5 and 33.2°C for *P. muralis* and *I. horvathi*, respectively) (Osojnik et al., 2013). Active individuals were forced to maintain body temperatures close to preferred body temperatures (spring: *T*_pref_ = 26.9 and 27.8°C; summer: *T*_pref_ = 31.2 and 30.4°C for *P. muralis* and *I. horvathi*; Osojnik et al., 2013). To account for seasonal variability, we ran the models with two sets of parameters. The two sets of parameters were different only in the values based on previously reported seasonal variability for *T*_pref_, *T*_forage_min_ and *T*_forage_max_ (Osojnik et al., 2013). When body temperatures exceeded *T*_forage_max_ or *T*_forage_min_, we simulated the use of shelters in the substrate at a depth where the temperature did not go out of bounds of the animals' critical thermal minima/maxima (5.1°C/43.0°C for *P. muralis* and 5.7°C/42.2°C for *I. horvathi*; Bennett et al., 2018; Bodensteiner et al., 2021; Herrando‐Pérez et al., 2020). For adults, we set the reproductive period between spring and summer, starting with a photoperiod of 12.6 h of sunlight and ending at 14 h of sunlight roughly corresponding to late March and early September, respectively, for the simulated localities. Size‐dependent litter production predictions made during the DEB modelling were further refined by using a linear relationship between snout–vent length and clutch size obtained from Galeotti et al. (2013) for *P. muralis* and using data from a previous study on *I. horvathi* reproduction in Slovenia (Ljubisavljević et al., 2012). It is noteworthy that feeding only occurred when animals were predicted to be active above ground, and we assumed that food was available ad libitum. We ran 30 separate biophysical models (15 localities and two species) from which we extracted six life‐history traits: Egg development time, total lifespan, years of reproduction, yearly basking time, yearly foraging time and yearly fecundity. Activity times were calculated as the number of hours the animals were predicted to be active in 1 year. Behavioural predictions were assigned to two categories: ‘basking’ (*T*_b‐s_ between *T*_emerge_ and *T*_forage_min_), which is the time spent reaching body temperatures that allow ‘foraging’, and ‘foraging’ (*T*_b‐s_ between *T*_forage_min_ and *T*_forage_max_) related to feeding, mating, etc. We additionally extracted data on location‐specific times without snow cover and mean daily maximum solar radiation received by the simulated lizard.

### 2.4 Statistical analysis

All analyses and visualizations were carried out in R v4.4.1 (R Core Team, 2024). We used several tidyverse (Wickham et al., 2019) functions to prepare the data for final analysis. To identify differences in microclimatic conditions that could determine lizards' life history and, thus, influence the patterns of coexistence, we built ordinary least squares regressions between obtained microclimatic estimates, elevation and location type. Location type was the categorical factor describing syntopy/allotopy. More precisely, the ordinary least squares (OLS) regressions took the form: variable ~ elevation + location type. In addition, we constructed models with interaction terms between predictors, but no interaction resulted significant, and interaction terms were removed. We compared the models using AICc where simpler models were favoured (ΔAICc >2) (Hurvich & Tsai, 1989; Mazerolle, 2023). Second, we compared the ectotherm model outputs between elevation, species and the three location types. We tested the effects of all three independent variables by constructing OLS models including pairwise interaction terms for each dependent variable separately with the syntax: variable ~ Elevation × Species × Location type. We again compared the models by AICc and opted for simpler models (without interactions) unless we observed a significant interaction and removed the interaction terms where less complex models were favoured. All the constructed models were checked for normality of residuals, homoscedasticity and additionally visually checked for normality using Q‐Q plots. Since we calculated individual coefficient results for the OLS models using the summary() function which assesses individual coefficients against the reference group, we additionally performed pairwise post hoc tests using Tukey tests as implemented in the glht() function of the multcomp R package (Hothorn et al., 2008) to account for possible pairwise differences in the effect of location type. We report the results for models constructed using spring and summer *T*_pref_ values.

## 3 RESULTS

### 3.1 Microclimatic conditions across elevations

**FIGURE 2:**
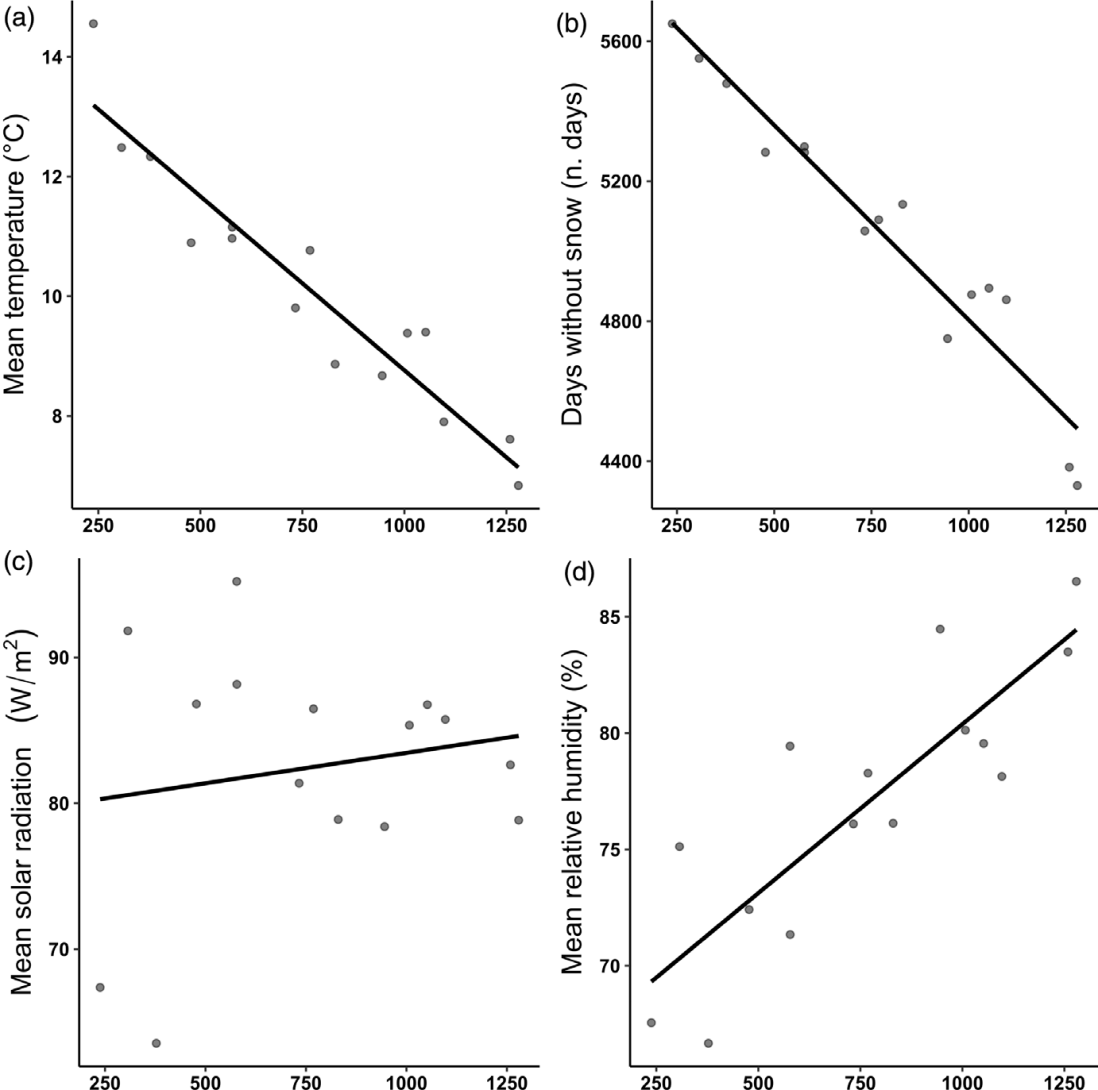
Plots of OLS linear regression models of the effect of elevation (*x* axis) on chosen environmental variables at 15 different locations of known allotopy or syntopy. Each point represents a unique location. Each data point in (a, c, d) represents the average mean yearly temperature during our simulation (16 years). The points in (b) represent the total number of days without snow in the full simulation.

All the analysed microclimatic variables except mean solar radiation significantly varied along the elevational gradient (Table S4; Figure 2). Mean ambient temperature decreased (*β* = −0.0054, *p* < 0.001) while relative mean humidity (*β* = 0.015, *p* < 0.001) increased with elevation. The yearly number of days without snow during the whole simulation of the animal's life decreased with elevation (*β* = −1.053, *p* < 0.001). There was no significant change with elevation in mean yearly solar radiation (*β* = 0.003, *p* = 0.773). Importantly, we found no significant differences in microclimatic variables between the three location types (Table S4; Figure 2).

### 3.2 Life‐history traits

**FIGURE 3:**
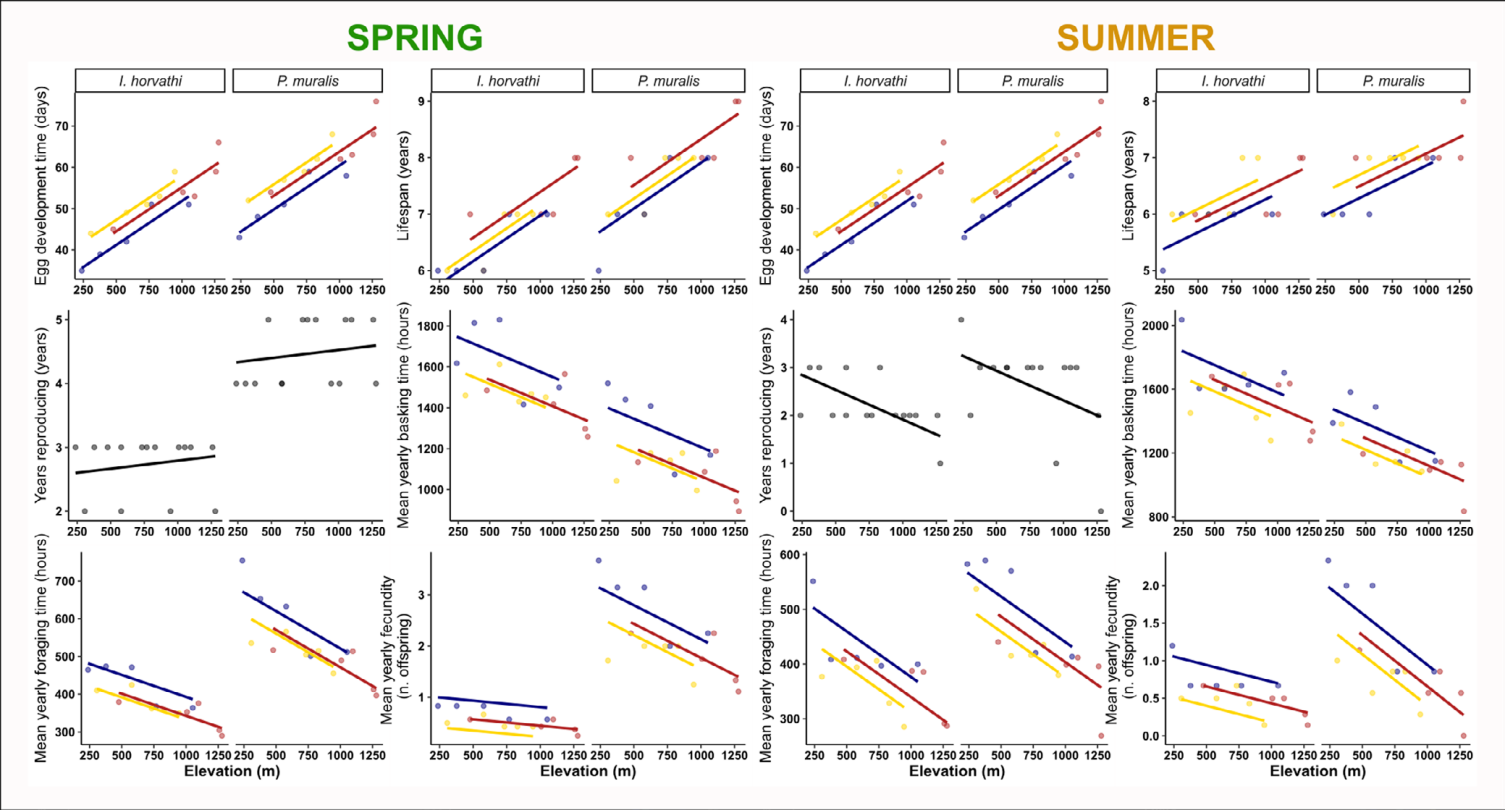
Plots of OLS linear regressions of the effect of elevation, species and location type on life‐history traits (blue—syntopy study sites, red—highland species *Iberolacerta horvathi* allotopy study sites, yellow—lowland species *Podarcis muralis* allotopy study sites) at 30 different microclimate–species trait combinations. Panels on the left represent simulation predictions when using spring preferred body temperatures and panels on the right when using summer preferred body temperatures. In the case of years reproducing, we avoided showing separate slopes by location type (syntopy/allotopy) since we found no significant effect. Yearly fecundity is expressed as mean yearly fecundity throughout the reproductive years of the animal.

The predictions from our models show significant differences between the two species in their simulated life‐history traits (Tables S5–S8). *Podarics muralis* had longer egg development times (using *T*_pref_ measured in spring and in summer: *β* = 8.53, *p* < 0.001) as well as longer lifespans (spring *T*_pref_: *β* = 0.93, *p* < 0.01; summer *T*_pref_: *β* = 0.60, *p* < 0.01) and more reproductive years (only spring *T*_pref_: *β* = 1.73, *p* < 0.001) than *I. horvathi* (Figure 3). *Podarcis muralis* spent less time basking (spring *T*_pref_: *β* = −348, *p* < 0.001; summer *T*_pref_: *β* = −365, *p* < 0.001) and had more available time for foraging (spring *T*_pref_: *β* = 0210, *p* < 0.001; summer *T*_pref_: *β* = 63.8, *p* < 0.001) (Figure 3). We observed no effect of species on reproductive years when using summer *T*_pref_ (*β* = 0.40, *p* = 0.128).

We also found strong effects of elevation on all simulated life‐history traits. Longer egg development times (spring/summer *T*_pref_: *β* = 0.02, *p* < 0.001) as well as longer lifespans (spring *T*_pref_: *β* = 0.002, *p* < 0.01; summer *T*_pref_: *β* = 0.001, *p* < 0.01) were observed at higher elevations (Figure 3). Increasing elevation negatively influenced the number of years the animals reproduced (summer *T*_pref_: *β* = −0.001, *p* < 0.01) as well as the time available for animals to bask (spring *T*_pref_: *β* = 0.26, *p* < 0.01; summer *T*_pref_: *β* = −0.35, *p* < 0.01) and forage (spring *T*_pref_: *β* = 0.12, *p* < 0.01; summer *T*_pref_: *β* = −0.16, *p* < 0.01) (Figure 3). There was a statistically significant negative interaction between elevation and species on yearly fecundity: elevation impacted more negatively the fecundity of *P. muralis* than *I. horvathi* (spring *T*_pref_: *β* = 0.001, *p* < 0.01; summer *T*_pref_: *β* = −0.00091, *p* < 0.01) (Figure 3). We observed no effect of elevation on years reproducing when using spring *T*_pref_ values.

Finally, location type also showed some significant effects on life‐history traits (Tables S6 and S8). Egg development time was shorter in syntopic localities than in allotopic localities of *P. muralis* (spring *T*_pref_: *β* = −6.08, *p* < 0.001; summer *T*_pref_: *β* = −6.17, *p* < 0.001) (Figure 3). Time spent basking and foraging differed between syntopic localities and *P. murallis* allotopy. Lizards had more time to bask (spring *T*_pref_: *β* = 163, *p* < 0.01; summer *T*_pref_: *β* = 165, *p* < 0.05) as well as more time to forage (spring *T*_pref_: *β* =59.5, *p* < 0.001; summer *T*_pref_: *β* = 64.4, *p* < 0.005) in syntopy (Figure 3). We also found significant differences between syntopic and *P. muralis* allotopic localities in mean yearly fecundity, with syntopic localities being more productive (spring *T*_pref_: *β* = 0.58, *p* < 0.001; summer *T*_pref_: *β* = 0.54, *p* < 0.001) (Figure 3). Additionally, we observed no significant difference in lifespan when using summer *T*_pref_, indicating a trend of lifespans being shorter in syntopic localities than in *P. muralis* allotopy (spring *T*_pref_: *β* = −0.18, *p* = 0.52; summer *T*_pref_: *β* = −0.41, *p* < 0.05) (Figure 3). We observed no significant effects of location types on the number of years reproducing. Interestingly, our post hoc tests revealed no significant differences between syntopic and *I. horvathi* allotopic sites.

## 4 DISCUSSION

Here, we set out to offer a novel approach to addressing a long‐standing question in ecology: which factors promote coexistence among species along environmental gradients? An integrated mechanistic modelling approach using DEB theory and NicheMapR was applied to a two‐species system to derive the relative role of abiotic factors and biotic interactions in explaining species distribution patterns. Our results point in the direction that abiotic factors alone do not explain current species ranges and spatial overlap patterns in *Podarcis muralis* and *Iberolacerta horvathi*. We found that, when compared with allotopic *P. muralis* locations, syntopic locations provide more favourable abiotic conditions through high microclimatic variability that promotes higher fecundity, activity and faster development, which might facilitate species coexistence. However, it is difficult to exclude the possibility of historical contingencies playing a role in the observed spatial distribution along the elevational gradient since both species exhibit specific adaptations to either lowland or highland conditions that may be due to evolutionary processes. Moreover, in our study area, the sites with *P. muralis* at the highest elevation might represent the elevation limit of this species here, while this species is known to occur at higher elevations elsewhere in its range (e.g. Gangloff et al., 2019) where we could expect differences in historical contingencies and specific adaptations (e.g. thermal preferences).

While our study species are generally adapted to either highlands (*I. horvathi*) or lowlands (*P. muralis*), we expected that our models would capture different responses in their life‐history traits across elevation. It is well known that temperature, as well as other abiotic factors such as humidity and solar radiation, varies across elevational gradients and is one of the main factors posing biophysical constraints on ectotherm life (Shine, 2005). In our system, ambient temperature and the number of days without snow decrease with elevation, while solar radiation and humidity showed an opposite trend. Lower temperatures and prolonged periods of snow at higher elevations are known to impact lizard maturation times (Angilletta et al., 2004), while studies looking explicitly at ambient temperature consistently show that ectotherms in cold environments live longer (Flouris & Piantoni, 2014). In agreement, our simulations showed that animals at higher elevations experience a delay both in egg development times as well as in general development, resulting in a prolonged lifespan.

High elevations may pose thermal constraints over ectotherms (Navas, 2002; Sunday et al., 2014). To overcome these limitations, ectotherms such as lizards may use behavioural thermoregulation to ensure that body temperature is maintained within favourable values (Pearson & Bradford, 1976; Smith & Ballinger, 1994). Previous studies have demonstrated that, in heliothermic ectotherms, the proportion of time spent basking versus foraging typically increases with lower ambient temperatures (Vitt & Caldwell, 2013). Contrary to this, our results reveal a significant decrease in both forms of activity (basking and foraging) with increasing elevation where the combination of lower temperatures and longer snow cover limits the activity windows of lizards. However, our simulations suggested that species differ—the highland *I. horvathi* spends more time thermoregulating than lowland *P. muralis*, and *I. horvathi* spends less time foraging than *P. muralis* at high elevations. This result suggests that *I. horvathi* probably spends more time than *P. muralis* on behavioural thermoregulation. This is in line with a previous study that showed narrower thermal preferences in *I. horvathi* when compared with *P. muralis* (Osojnik et al., 2013). However, whether the field‐active lizards of both species reach different body temperatures has not yet been measured and could be tested by further targeted studies to verify the model predictions. A further important aspect of thermoregulation that should be investigated through targeted experimental studies is the efficiency of thermoregulation. These data could in turn be used to fine‐tune the mechanistic models.

Moreover, more time spent basking also means a higher risk of predation. Therefore, highland species should have developed a better predator avoidance strategy, which has already been confirmed by an empirical study showing that *I. horvathi* escapes to shelter earlier than *P. muralis* when reacting to an approaching individual (Žagar, Bitenc, et al., 2015). Furthermore, empirical data suggested that *I. horvathi* has higher metabolic rates and faster metabolism (higher potential metabolic activity; Žagar, Simčič, et al., 2015) than *P. muralis*. Higher metabolic rates represent greater energy expenditure, which needs to be fuelled either by greater energy intake (i.e. increased foraging times; Huey & Pianka, 1981) or greater efficiency in energy assimilation (i.e. animals eat less but can maximize energy intake; Somero et al., 2017). Our simulations predicted that highland species (*I. horvathi*) exhibited lower foraging times than its lowland counterpart due to spending more time basking. It is possible that the metabolic efficiency of *I. horvathi* is higher (which is supported to some extent by previous research that found higher aerobic respiration efficiency; Žagar, Simčič, et al., 2015), or that the prey capture efficiency is higher than that of *P. muralis* to compensate for the shorter foraging time. However, the latter would need to be verified by empirical studies in the future.

Our simulations predicted faster egg development in *I. horvathi* than in *P. muralis*. Clutch sizes and their frequency are known to vary by elevation. An example in passerine birds showed that clutch size generally decreases with increasing altitude (Hille et al., 2014), while a study looking at lizards found that annual temperatures positively correlate with clutch frequency and negatively correlate with clutch size (Meiri et al., 2013). Our model predicted that the fecundity of the lowland species decreases faster with increasing elevation than the fecundity of the highland species. The ability of *I. horvathi* to maintain stable yearly fecundity in colder environments likely reflects the adaptation of this species to high‐altitude environment. The yearly fecundity of *P. muralis* declined steeply with increasing elevation reaching a point of no successful reproduction at the highest elevations (1279 m); this suggests that the reproductive capacity of the lowland species is due to adaptation to the warmer climate in the lowlands. Data from other regions of the distribution of *P. muralis* confirm that these animals can successfully reproduce even at much higher elevations (i.e. up to 2400 m above sea level), but they do experience sex‐specific abiotic limitations leading to smaller male body sizes and lower female body condition at higher elevations (Perry et al., 2024). This suggests that local adaptation may play a role in shaping the life‐history traits of this species, and examination of other parts of the species' range where they have a greater altitudinal distribution span may yield different results. Our results on predicted fecundity suggest that high elevations in our study site may exhibit a combination of microclimatic factors that limit the distribution of lowland species.

Seasonal changes in *T*_pref_ used in our models influenced some life‐history traits such as lifespan and reproductive output and impacted the results on the number of years reproducing. These findings align with the understanding that preferred body temperature is an important factor that changes seasonally and therefore has a seasonally variable impact on ectotherm physiology, performance and life history (Giacometti et al., 2024). Additionally, seasonality can be an important driver of physiological states such as pregnancy which can by itself influence preferred body temperatures (Mathies & Andrews, 1997). Furthermore, it has been shown that a certain level of plasticity in *T*_pref_ is crucial to maintain fitness across seasons (Angilletta, 2009). These and our findings show the importance of seasonal variation in thermal preferences in ectotherms to adapt to regular cycles of differing abiotic thermal conditions. The observed differences in life‐history traits when using spring and summer thermal preferences in our study further highlight the importance of integrating seasonally shifting thermal traits with life history and ecological data to advance our understanding of ectotherm thermoregulation strategies (Giacometti et al., 2024). Approaches such as ours using mechanistic models are a great way to integrate these various levels of data at the individual level and offer a promising approach to study how seasonality affects ectotherms on both a micro‐ and macroecological scale.

Elevational segregation patterns of competing species are known from a variety of taxa (e.g. Chan et al., 2019; Tannerfeldt et al., 2002; Vrezec & Tome, 2004). For example, Bastianelli et al. (2017) used a modelling approach to study bird species turnover, where they concluded that no single mechanism drove the distribution of their coexisting species, but the distributions were defined by many small forces including both biotic and abiotic factors. Previous research supports the idea that competition might be a driving force in our study system (Žagar, Carretero, et al., 2015), as well as in other closely related species (e.g. Carranza et al., 2004). By comparing microclimate and life‐history traits between sites of allotopy and syntopy, we were able to gain insight into the effects of potential exclusion between species, which is an indirect way of looking for evidence of competition (Pianka, 1981). While our microclimatic models did not capture any significant variation between location types, we did observe important differences in life‐history traits (i.e. after running the ectotherm model). Thus, these results imply that the observed differences are not due to microclimates alone but are an interaction between abiotic factors and species traits. Moreover, in syntopy, both species exhibit lower developmental times and lifespan, higher basking and activity times as well as higher fecundity. Importantly, we only observed significant pairwise differences between syntopy and allotopic P. *muralis*. Observing that both species reach their highest fecundities in syntopic localities is interesting when viewed through contemporary coexistence theory (Chesson, 2000). This theory posits that intraspecific density dependence should be stronger than interspecific (Chesson, 2000). Consequently, higher population densities of each species can lead to increased intraspecific competition, which in turn results in competitive release for interspecific competition (Alatalo et al., 1985).Furthermore, it seems that the syntopic sites which mainly occur at middle elevations are extremely favourable for both species. The animals attain high metabolic rates, reproduce more and grow fast in syntopic sites, which favours the abundance hypothesis representing syntopy as the most favourable conditions where species share abundant environmental (abiotic) resources (Brodie et al., 2018). It is worth noting that maintaining a higher metabolic rate is only beneficial when resources are abundant; otherwise, energy stores are quickly depleted, leading to lethargy or even death. Previous studies have already empirically shown changes in relative abundance across elevation (Žagar, 2016) which, when combined with our results, lead us to conclude that, in our system, allotopy may be a function of competitive exclusion under suboptimal conditions for one or the other species (Means, 1975; Reif et al., 2018) with *P. muralis* being excluded at high elevations and a more complex picture of coexistence of species in the lowlands. Additionally, syntopy possibly represents a case of abiotic variability leading to the promotion of coexistence (Chesson, 2000; Cloyed & Eason, 2017). Additionally, we conclude that it is essential to include both abiotic data as well as species traits when studying species spatial separation.

In the quest to understand the factors that favour coexistence of species along environmental gradients, we have demonstrated that the interplay between abiotic factors and species traits is a cornerstone. It is tempting to assume that abiotic factors are responsible for the spatial distribution of ecologically similar species along environmental gradients (e.g. Gaston, 2003) or that historical/geographical factors play a role (e.g. Wiens & Donoghue, 2004). Regarding the influence of historical contingencies, it is worth mentioning that obtaining distribution data from biogeographic studies might turn out to be crucial (de Carvalho et al., 2013). However, we show that more complex, interacting mechanisms may underlie species coexistence patterns along climatic gradients. In this sense, we show that the integration of abiotic conditions and species functional traits is key if we want to predict the spatial distribution and overlap of species. Furthermore, with our study, we exemplify that our mechanistic modelling‐based approach offers essential tools to advance understanding of patterns of coexistence in ecologically similar species even when considering its obvious limitation of heavy reliance on input data and complex modelling procedures. Future work on this topic should focus on facilitating the integration of empirical, experimental and field studies with mechanistic model approaches.

## AUTHOR CONTRIBUTIONS

Urban Dajčman and Anamarija Žagar proposed the original idea; Urban Dajčman, Anamarija Žagar and Urtzi Enriquez‐Urzelai assembled the database; Urban Dajčman and Urtzi Enriquez‐Urzelai analysed the data; Urban Dajčman wrote the first draft of the manuscript and prepared the visualizations. All authors contributed substantially to revisions. All authors agreed on the final approval of the version to be published and agreed that they are accountable for the work they conducted.

## CONFLICT OF INTEREST STATEMENT

We declare that none of the authors have any actual or potential conflict of interest, including any financial, personal or other relationships with other people or organizations that could inappropriately influence or be perceived to influence our work.

## STATEMENT ON INCLUSION

Our research unites contributors from various countries, featuring scientists located in the nation where the research was conducted. These authors were involved from the beginning in both the research and its design, to incorporate the varied viewpoints they offer right from the start. When appropriate, we cited studies by regional scientists and made a concerted effort to acknowledge significant research published in the native language.

## Supporting information


**Figure S1.** Schematic representation of the metabolic processes in the standard DEB model.
**Figure S2.** Plots of univariate data used in DEB model estimation.
**Table S1.** Life history data and corresponding model predictions for the two studied lizard species: Horvath's rock lizard (*Iberolacerta horvathi*) and Common wall lizard (*Podarcis muralis*).
**Table S2.** Table used to estimate temperatures at start of activity for both species of lizards.
**Table S3.** Core DEB parameters for the two studied species of lizards.
**Table S4.** Individual coefficient results of OLS linear regression models of the effect of elevation and location type on environmental variables.
**Table S5.** Individual coefficient results of linear models of the effect of elevation, species, and location type on life history traits when modelled using spring preferred body temperatures.
**Table S6.** Post hoc comparisons of location types when modelled using spring preferred body temperatures.
**Table S7.** Individual coefficient results of linear models of the effect of elevation, species, and location type on life history traits when modelled using summer preferred body temperatures.
**Table S8.** Post hoc comparisons of location types when modelled using summer preferred body temperatures.

**Data S1.** R code examples

## Data Availability

Full data and R scripts used for modelling and data analysis are available at the Zenodo digital repository (Dajčman et al., 2024, https://doi.org/10.5281/zenodo.10830164).

## References

[jane70030-bib-1002] Adolph, S. C. , & Porter, W. P. (1996). Growth, seasonality, and lizard life histories: Age and size at maturity. Oikos, 77(2), 267. 10.2307/3546065

[jane70030-bib-0001] Alatalo, R. V. , Gustafsson, L. , Lundberg, A. , & Ulfstrand, S. (1985). Habitat shift of the Willow Tit Parus montanus in the absence of the Marsh Tit Parus palustris. Ornis Scandinavica, 16, 121–128. 10.2307/3676477

[jane70030-bib-1006] AmPtool . (2022). Software package AmPtool. https://github.com/add‐my‐pet/AmPtool

[jane70030-bib-0002] Anderson, R. O. , Alton, L. A. , White, C. R. , & Chapple, D. G. (2022). Ecophysiology of a small ectotherm tracks environmental variation along an elevational cline. Journal of Biogeography, 49(2), 405–415. 10.1111/jbi.14311

[jane70030-bib-0003] Angilletta Jr, M. J. , Sears, M. W. , Levy, O. , Youngblood, J. P. , & Van den Brooks, J. M. (2019). Fundamental flaws with the fundamental niche. Integrative and Comparative Biology, 59(4), 1038–1048. 10.1093/icb/icz084 31141123

[jane70030-bib-0004] Angilletta, M. J. (2009). Thermal adaptation: A theoretical and empirical synthesis. Oxford University Press.

[jane70030-bib-0005] Angilletta, M. J. , Steury, T. D. , & Sears, M. W. (2004). Temperature, growth rate, and body size in ectotherms: Fitting pieces of a life‐history puzzle. Integrative and Comparative Biology, 44(6), 498–509. 10.1093/icb/44.6.498 21676736

[jane70030-bib-0006] Arribas, O. , & Galán, P. (2005). Reproductive characteristics of the Pyrenean high‐mountain lizards: *Iberolacerta aranica* (Arribas, 1993), I. Aurelioi (Arribas, 1994) and I. Bonnali (Lantz, 1927). Animal Biology, 55(2), 163–190. 10.1163/1570756053993505

[jane70030-bib-0007] Arrizabalaga‐Escudero, A. , Clare, E. L. , Salsamendi, E. , Alberdi, A. , Garin, I. , Aihartza, J. , & Goiti, U. (2018). Assessing niche partitioning of co‐occurring sibling bat species by DNA metabarcoding. Molecular Ecology, 27(5), 1273–1283. 10.1111/mec.14508 29411450

[jane70030-bib-1016] Bastianelli, G. , Wintle, B. A. , Martin, E. H. , Seoane, J. , & Laiolo, P. (2017). Species partitioning in a temperate mountain chain: Segregation by habitat vs. interspecific competition. Ecology and Evolution, 7(8), 2685–2696. 10.1002/ece3.2883 28428859 PMC5395447

[jane70030-bib-0008] Begon, M. , & Townsend, C. R. (2021). Ecology: From individuals to ecosystems (5th ed.). Wiley.

[jane70030-bib-0009] Bennett, J. , Calosi, P. , Clusella‐Trullas, S. , Martínez, B. , Sunday, J. , Algar, A. C. , Araújo, M. B. , Hawkins, B. A. , Keith, S. , Kühn, I. , Rahbek, C. , Rodríguez, L. , Singer, A. , Villalobos, F. , Olalla‐Tárraga, M. Á. , & Morales‐Castilla, I. (2018). GlobTherm, a global database on thermal tolerances for aquatic and terrestrial organisms. Scientific Data, 5, 180022. 10.1038/sdata.2018.22 29533392 PMC5848787

[jane70030-bib-0010] Bertness, M. D. , & Callaway, R. (1994). Positive interactions in communities. Trends in Ecology & Evolution, 9(5), 191–193. 10.1016/0169-5347(94)90088-4 21236818

[jane70030-bib-0011] Bodensteiner, B. L. , Gangloff, E. J. , Kouyoumdjian, L. , Muñoz, M. M. , & Aubret, F. (2021). Thermal–metabolic phenotypes of the lizard Podarcis muralis differ across elevation, but converge in high‐elevation hypoxia. Journal of Experimental Biology, 224(24), jeb243660. 10.1242/jeb.243660 34761802

[jane70030-bib-0012] Briscoe, N. J. , Elith, J. , Salguero‐Gómez, R. , Lahoz‐Monfort, J. J. , Camac, J. S. , Giljohann, K. M. , Holden, M. H. , Hradsky, B. A. , Kearney, M. R. , McMahon, S. M. , Phillips, B. L. , Regan, T. J. , Rhodes, J. R. , Wintle, B. A. , Yen, J. D. L. , Guillera‐Arroita, G. , & Vesk, P. A. (2019). Forecasting species range dynamics with process‐explicit models: Matching methods to applications. Ecology Letters, 22(11), 1940–1956. 10.1111/ele.13348 31359571

[jane70030-bib-0013] Briscoe, N. J. , Morris, S. D. , Mathewson, P. D. , Buckley, L. B. , Jusup, M. , Levy, O. , Maclean, I. M. D. , Pincebourde, S. , Riddell, E. A. , Roberts, J. A. , Schouten, R. , Sears, M. W. , & Kearney, M. R. (2023). Mechanistic forecasts of species responses to climate change: The promise of biophysical ecology. Global Change Biology, 29(6), 1451–1470. 10.1111/gcb.16557 36515542

[jane70030-bib-0014] Brodie, J. F. , Helmy, O. E. , Mohd‐Azlan, J. , Granados, A. , Bernard, H. , Giordano, A. J. , & Zipkin, E. (2018). Models for assessing local‐scale co‐abundance of animal species while accounting for differential detectability and varied responses to the environment. Biotropica, 50, 5–15. 10.1111/btp.12500

[jane70030-bib-0015] Brown, J. L. , & Carnaval, A. C. (2019). A tale of two niches: Methods, concepts, and evolution. Frontiers of Biogeography, 11(4), e44158. 10.21425/F5FBG44158

[jane70030-bib-0016] Buckley, L. B. , & Roughgarden, J. (2006). Climate, competition, and the coexistence of Island lizards. Functional Ecology, 20, 315–322. 10.1111/j.1365-2435.2006.01095.x

[jane70030-bib-0017] Cabela, A. , Grillitsch, H. , & Tiedemann, F. (2007). Habitatpräferenzen von *Podarcis muralis* (Laurenti, 1768) und *Iberolacerta horvathi* (Méhely, 1904) bei gemeinsamem Vorkommen. Herpetozoa, 19(3/4), 149–160.

[jane70030-bib-0018] Caldwell, A. J. , While, G. M. , & Wapstra, E. (2017). Plasticity of thermoregulatory behaviour in response to the thermal environment by widespread and alpine reptile species. Animal Behaviour, 132, 217–227. 10.1016/j.anbehav.2017.07.025

[jane70030-bib-0019] Carranza, S. , Arnold, E. N. , & Amat, F. (2004). DNA phylogeny of *Lacerta* (*Iberolacerta*) and other lacertine lizards (Reptilia: Lacertidae): Did competition cause long‐term mountain restriction? Systematics and Biodiversity, 2(1), 57–77. 10.1017/S1477200004001355

[jane70030-bib-0020] Castanet, J. (1994). Age estimation and longevity in reptiles. Gerontology, 40, 174–192. 10.1159/000213586 7926855

[jane70030-bib-0021] Castanet, J. , & Roche, E. (1981). Détermination de l'âge chez le lézard des murailles, *Lacerta muralis* (Laurenti, 1768) au moyen de la squelettochronologie. Revue Suisse de Zoologie, 88, 215–226. 10.5962/bhl.part.82365

[jane70030-bib-0022] Chan, S. F. , Shih, W. K. , Chang, A. Y. , Shen, S. F. , & Chen, I. C. (2019). Contrasting forms of competition set elevational range limits of species. Ecology Letters, 22(10), 1668–1679. 10.1111/ele.13342 31347240

[jane70030-bib-0023] Chesson, P. (2000). Mechanisms of maintenance of species diversity. Annual Review of Ecology and Systematics, 31(1), 343–366. 10.1146/annurev.ecolsys.31.1.343

[jane70030-bib-0024] Chesson, P. , & Huntly, N. (1997). The roles of harsh and fluctuating conditions in the dynamics of ecological communities. The American Naturalist, 150(5), 519–553. 10.1086/286080 18811299

[jane70030-bib-0025] Cloyed, C. S. , & Eason, P. K. (2017). Niche partitioning and the role of intraspecific niche variation in structuring a guild of generalist anurans. Royal Society Open Science, 4(3), 170060. 10.1098/rsos.170060 28405403 PMC5383860

[jane70030-bib-0026] Clusella‐Trullas, S. , Blackburn, T. M. , & Chown, S. L. (2011). Climatic predictors of temperature performance curve parameters in ectotherms imply complex responses to climate change. The American Naturalist, 177(6), 738–751. 10.1086/660021 21597251

[jane70030-bib-0027] Colwell, R. K. , & Lees, D. C. (2000). The mid‐domain effect: Geometric constraints on the geography of species richness. Trends in Ecology & Evolution, 15(2), 70–76. 10.1016/S0169-5347(99)01767-X 10652559

[jane70030-bib-0028] Dajčman, U. , Carretero, M. A. , Megía‐Palma, R. , Perera, A. , & Žagar, A. (2022). Shared haemogregarine infections in competing lacertids. Parasitology, 149(2), 193–202. 10.1017/S0031182021001645 35234602 PMC11010482

[jane70030-bib-0029] Dajčman, U. , Enriquez‐Urzelai, U. , & Žagar, A. (2024). Data for: Microclimate variability impacts the coexistence of highland and lowland ectotherms. *Zenodo Digital Repository*. 10.5281/zenodo.10830164 PMC12056348 40108979

[jane70030-bib-1007] DEBtool . (2022). Software package DEBtool_M. https://github.com/add‐my‐pet/DEBtool_M

[jane70030-bib-0030] de Carvalho, A. L. G. , de Britto, M. R. , & Fernandes, D. S. (2013). Biogeography of the lizard genus *Tropidurus* Wied‐Neuwied, 1825 (Squamata: Tropiduridae): Distribution, endemism, and area relationships in South America. PLoS One, 8(3), e59736. 10.1371/journal.pone.0059736 23527261 PMC3602109

[jane70030-bib-0031] De Luca, N. (1989). Taxonomic and biogeographic characteristics of Horvath's rock lizard (Lacerta horvathi Méhely, 1904, Lacertidae, Reptilia) in Yugoslavia. Scopolia, 18, 1–48.

[jane70030-bib-0032] Elton, C. S. (1927). Animal ecology. University of Chicago Press. 10.1046/j.1365-2435.1997.00058.x

[jane70030-bib-0033] Eroğlu, A. İ. , Bülbül, U. , Kurnaz, M. , & Odabaş, Y. (2018). Age and growth of the common wall lizard, *Podarcis muralis* (Laurenti, 1768). Animal Biology, 68(2), 147–159. 10.1163/15707563-17000019

[jane70030-bib-0034] Flouris, A. D. , & Piantoni, C. (2014). Links between thermoregulation and aging in endotherms and ectotherms. Temperature, 2(1), 73–85. 10.4161/23328940.2014.989793 PMC4843886 27226994

[jane70030-bib-1009] Galeotti, P. , Sacchi, R. , Pellitteri‐Rosa, D. , Bellati, A. , Cocca, W. , Gentilli, A. , Scali, S. , & Fasola, M. (2013). Colour polymorphism and alternative breeding strategies: Effects of Parent's colour morph on fitness traits in the common wall lizard. Evolutionary Biology, 40(3), 385–394. 10.1007/s11692-012-9222-3

[jane70030-bib-0035] Gangloff, E. J. , Sorlin, M. , Cordero, G. A. , Souchet, J. , & Aubret, F. (2019). Lizards at the peak: Physiological plasticity does not maintain performance in lizards transplanted to high altitude. Physiological and Biochemical Zoology, 92(2), 189–200. 10.1086/701793 30714846

[jane70030-bib-0036] Gaston, K. J. (2003). The structure and dynamics of geographic ranges. Oxford University Press.

[jane70030-bib-0037] Gause, G. F. (1934). The struggle for existence. The Williams and Wilkins Company.

[jane70030-bib-0038] Giacometti, D. , Bars‐Closel, M. , Kohlsdorf, T. , de Carvalho, J. E. , & de Cury Barros, F. (2022). Environmental temperature predicts resting metabolic rates in tropidurinae lizards. Journal of Experimental Zoology Part A: Ecological and Integrative Physiology, 337(9–10), 1039–1052. 10.1002/jez.2656 36127811

[jane70030-bib-0039] Giacometti, D. , Palaoro, A. V. , Leal, L. C. , & de Barros, F. C. (2024). How seasonality influencesthe thermal biology of lizards with different thermoregulatory strategies: A meta‐analysis. Biological Reviews, 99(2), 409–429. 10.1111/brv.13028 37872698

[jane70030-bib-0040] Gravel, D. , Guichard, F. , & Hochberg, M. E. (2011). Species coexistence in a variable world. Ecology Letters, 14(8), 828–839. 10.1111/j.1461-0248.2011.01643.x 21682832

[jane70030-bib-0041] Gravel, D. , & Massol, F. (2020). Toward a general theory of metacommunity ecology. In K. S. McCann & G. Gellner (Eds.), Theoretical ecology: Concepts and applications. Oxford University Press. 10.1093/oso/9780198824282.003.0012

[jane70030-bib-0042] Grigg, J. W. , & Buckley, L. B. (2013). Conservatism of lizard thermal tolerances and body temperatures across evolutionary history and geography. Biology Letters, 9(2), 20121056. 10.1098/rsbl.2012.1056 23325735 PMC3639756

[jane70030-bib-1001] Grinnell, J. (1917). The niche‐relationships of the California Thrasher. The Auk, 34(4), 427–433. 10.2307/4072271

[jane70030-bib-0043] Guisan, A. , & Thuiller, W. (2005). Predicting species distribution: Offering more than simple habitat models. Ecology Letters, 8, 993–1009. 10.1111/j.1461-0248.2005.00792.x 34517687

[jane70030-bib-0044] Hengl, T. , de Mens Jesus, J. , Heuvelink, G. B. , Ruiperez Gonzalez, M. , Kilibarda, M. , Blagotić, A. , Heuvelink, G. B. M. , Shangguan, W. , Wright, M. N. , Geng, X. , Bauer‐Marschallinger, B. , Guevara, M. A. , Vargas, R. , MacMillan, R. A. , Batjes, N. H. , Leenaars, J. G. B. , Ribeiro, E. , Wheeler, I. , Mantel, S. , & Kempen, B. (2017). SoilGrids250m: Global gridded soil information based on machine learning. PLoS One, 12(2), e0169748. 10.1371/journal.pone.0169748 28207752 PMC5313206

[jane70030-bib-0045] Herrando‐Pérez, S. , Monasterio, C. , Beukema, W. , Gomes, V. , Ferri‐Yáñez, F. , Vieites, D. R. , Buckley, L. B. , & Araújo, M. B. (2020). Heat tolerance is more variable than cold tolerance across species of Iberian lizards after controlling for intraspecific variation. Functional Ecology, 34, 631–645. 10.1111/1365-2435.13507

[jane70030-bib-0046] Herzog, S. K. , Kessler, M. , & Bach, K. (2005). The elevational gradient in Andean bird species richness at the local scale: A foothill peak and a high‐elevation plateau. Ecography, 28(2), 209–222. 10.1111/j.0906-7590.2005.03935.x

[jane70030-bib-1013] Hille, S. M. , & Cooper, C. B. (2014). Elevational trends in life histories: Revising the pace‐of‐life framework. Biological Reviews, 90(1), 204–213. 10.1111/brv.12106 24673806

[jane70030-bib-0047] Hollister, J. , Shah, T. , Robitaille, A. , Beck, M. , & Johnson, M. (2017). elevatr: access elevation data from various APIs. R package version 0.1, 3. U.S. EPA Office of Research and Development.

[jane70030-bib-1011] Hothorn, T. , Bretz, F. , & Westfall, P. (2008). Simultaneous inference in general parametric models. Biometrical Journal, 50(3), 346–363. 10.1002/bimj.200810425 18481363

[jane70030-bib-0048] Hubbell, S. P. (2001). The unified neutral theory of biodiversity and biogeography. Princeton University Press. 10.1016/j.tree.2011.03.024 21561679

[jane70030-bib-0049] Huey, R. B. , & Pianka, E. R. (1981). Ecological consequences of foraging mode. Ecology, 62(4), 991–999. 10.2307/1936998

[jane70030-bib-0050] Hurvich, C. M. , & Tsai, C. L. (1989). Regression and time series model selection in small samples. Biometrika, 76(2), 297–307. 10.2307/2336663

[jane70030-bib-0051] Ji, X. , & Brana, F. (2000). Among clutch variation in reproductive output and egg size in the wall lizard (*Podarcis muralis*) from a lowland population of northern Spain. Journal of Herpetology, 34, 54–60. 10.2307/1565238

[jane70030-bib-0052] Kalnay, E. , Kanamitsu, M. , Kistler, R. , Collins, W. , Deaven, D. , Gandin, L. , Iredell, M. , Saha, S. , White, G. , Woollen, J. , Zhu, Y. , Chelliah, M. , Ebisuzaki, W. , Higgins, W. , Janowiak, J. , Mo, K. C. , Ropelewski, C. , Wang, J. , Leetmaa, A. , … Joseph, D. (1996). The NCEP/NCAR 40‐year reanalysis project. Bulletin of the American Meteorological Society, 77, 437–471. 10.1175/1520-0477(1996)077<0437:TNYRP>2.0.CO;2

[jane70030-bib-0053] Kearney, M. (2012). Metabolic theory, life history and the distribution of a terrestrial ectotherm. Functional Ecology, 26(1), 167–179. 10.1111/j.1365-2435.2011.01917.x

[jane70030-bib-0054] Kearney, M. R. (2019). The fundamental niche concept connects individuals to populations: a comment on Angilletta et al. Integrative and Comparative Biology, 59(6), 1509–1510. 10.1093/icb/icz147 31397868

[jane70030-bib-0055] Kearney, M. , & Porter, W. (2009). Mechanistic niche modelling: Combining physiological and spatial data to predict species' ranges. Ecology Letters, 12(4), 334–350. 10.1111/j.1461-0248.2008.01277.x 19292794

[jane70030-bib-0057] Kearney, M. R. , & Porter, W. P. (2017). NicheMapR–an R package for biophysical modelling: The microclimate model. Ecography, 40(5), 664–674. 10.1111/ecog.02360

[jane70030-bib-1004] Kearney, M. R. , Gillingham, P. K. , Bramer, I. , Duffy, J. P. , & Maclean, I. M. D. (2019). A method for computing hourly, historical, terrain‐corrected microclimate anywhere on earth. Methods in Ecology and Evolution, 11(1), 38–43. 10.1111/2041-210x.13330

[jane70030-bib-0058] Kearney, M. R. , & Porter, W. P. (2019). NicheMapR – An R package for biophysical modelling: The ectotherm and Dynamic Energy Budget models. Ecography, 43(1), 85–96. 10.1111/ecog.04680

[jane70030-bib-0060] Kemp, M. U. , Van Loon, E. E. , Shamoun‐Baranes, J. , & Bouten, W. (2012). RNCEP: Global weather and climate data at your fingertips. Methods in Ecology and Evolution, 3(1), 65–70. 10.1111/j.2041-210X.2011.00138.x

[jane70030-bib-0061] Kooijman, B. (2009). Dynamic energy budget theory for metabolic organisation (3rd ed.). Cambridge University Press. 10.1017/CBO9780511805400

[jane70030-bib-0062] Krofel, M. , Cafuta, V. , Planinc, G. , Sopotnik, M. , Šalamun, A. , Tome, S. , Vamberger, M. , & Žagar, A. (2009). Razširjenost plazilcev v Sloveniji: pregled podatkov, zbranih do leta 2009. Natura Sloveniae, 11(2), 61–99. 10.14720/ns.11.2.61-99

[jane70030-bib-0063] Lapini, L. , Dall'Asta, A. , Luiselli, L. , & Nardi, P. (2004). Lacerto horvathi in Italy: A review with new data on distribution, spacing strategy and territoriality (Reptilia, Lacertidae). Bollettino di Zoologia, 71(S2), 145–151.

[jane70030-bib-0064] Lika, K. , Kearney, M. R. , Freitas, V. , van der Veer, H. W. , van der Meer, J. , Wijsman, J. W. , Pecquerie, L. , & Kooijman, S. A. (2011). The “covariation method” for estimating the parameters of the standard dynamic energy budget model I: Philosophy and approach. Journal of Sea Research, 66(4), 270–277. 10.1016/J.SEARES.2011.07.010

[jane70030-bib-0065] Ljubisavljević, K. , Glasnović, P. , Kalan, K. , & Kryštufek, B. (2012). Female reproductive characteristics of the Horvath's rock lizard (*Iberolacerta horvathi*) from Slovenia. Archives of Biological Sciences, 64(2), 639–645.

[jane70030-bib-0066] MacArthur, R. H. (1958). Population ecology of some warblers of northeastern coniferous forests. Ecology, 39(4), 599–619. 10.2307/1931600

[jane70030-bib-0067] MacArthur, R. H. (1984). Geographical ecology: Patterns in the distribution of species. Princeton University Press.

[jane70030-bib-0068] Maclean, I. M. , Mosedale, J. R. , & Bennie, J. J. (2019). Microclima: An r package for modelling meso‐and microclimate. Methods in Ecology and Evolution, 10(2), 280–290. 10.1111/2041-210X.13093

[jane70030-bib-0069] Maestre, F. T. , Callaway, R. M. , Valladares, F. , & Lortie, C. J. (2009). Refining the stress‐gradient hypothesis for competition and facilitation in plant communities. Journal of Ecology, 97(2), 199–205. 10.1111/j.1365-2745.2008.01476.x

[jane70030-bib-0070] Marn, N. , Hudina, S. , Haberle, I. , Dobrović, A. , & Klanjšček, T. (2022). Physiological performance of native and invasive crayfish species in a changing environment: Insights from dynamic energy budget models. Conservation Physiology, 10(1), coac031. 10.1093/conphys/coac031 35669378 PMC9156854

[jane70030-bib-0071] Marques, G. M. , Augustine, S. , Lika, K. , Pecquerie, L. , Domingos, T. , & Kooijman, S. A. (2018). The AmP project: Comparing species on the basis of dynamic energy budget parameters. PLoS Computational Biology, 14(5), e1006100. 10.1371/journal.pcbi.1006100 29742099 PMC5962104

[jane70030-bib-0072] Mathies, T. , & Andrews, R. M. (1997). Influence of pregnancy on the thermal biology of the lizard, *Scleroporus jarrovi*: Why do pregnant females exhibit low body temperatures? Functional Ecology, 11(4), 498–507. http://www.jstor.org/stable/2390385

[jane70030-bib-0073] Mazerolle, M. J. (2023). AICcmodavg: Model selection and multimodel inference based on (Q)AIC(c) (Version 2.3.3) [R package]. Comprehensive R Archive Network (CRAN). https://cran.r‐project.org/package=AICcmodavg

[jane70030-bib-0074] McCain, C. M. (2005). Elevational gradients in diversity of small mammals. Ecology, 86(2), 366–372. https://www.jstor.org/stable/3450957

[jane70030-bib-0075] Means, D. B. (1975). Competitive exclusion along a habitat gradient between two species of salamanders (*Desmognathus*) in western Florida. Journal of Biogeography, 2(4), 253–263. 10.2307/3037999

[jane70030-bib-1014] Meiri, S. , Bauer, A. M. , Chirio, L. , Colli, G. R. , Das, I. , Doan, T. M. , Feldman, A. , Herrera, F. , Novosolov, M. , Pafilis, P. , Pincheira‐Donoso, D. , Powney, G. , Torres‐Carvajal, O. , Uetz, P. , & Van Damme, R. (2013). Are lizards feeling the heat? A tale of ecology and evolution under two temperatures. Global Ecology and Biogeography, 22(7), 834–845. 10.1111/geb.12053

[jane70030-bib-0076] Meter, B. , Starostová, Z. , Kubička, L. , & Kratochvíl, L. (2020). The limits of the energetical perspective: Life‐history decisions in lizard growth. Evolutionary Ecology, 34, 469–481. 10.1007/s10682-020-10054-0

[jane70030-bib-0077] Meyer, A. V. , Sakairi, Y. , Kearney, M. R. , & Buckley, L. B. (2023). A guide and tools for selecting and accessing microclimate data for mechanistic niche modeling. Ecosphere, 14(4), e4506. 10.1002/ecs2.4506

[jane70030-bib-0078] Monasterio, C. , Shoo, L. P. , Salvador, A. , Siliceo, I. , & Díaz, J. A. (2011). Thermal constraints on embryonic development as a proximate cause for elevational range limits in two Mediterranean lacertid lizards. Ecography, 34(6), 1030–1039. 10.1111/j.1600-0587.2011.06905.x

[jane70030-bib-0079] Navas, C. A. (2002). Herpetological diversity along Andean elevational gradients: Links with physiological ecology and evolutionary physiology. Comparative Biochemistry and Physiology Part A: Molecular & Integrative Physiology, 133(3), 469–485. 10.1016/S1095-6433(02)00207-6 12443907

[jane70030-bib-0080] Nisbet, R. M. , Muller, E. B. , Lika, K. , & Kooijman, S. A. L. M. (2000). From molecules to ecosystems through dynamic energy budget models. Journal of Animal Ecology, 69(6), 913–926. 10.1111/j.1365-2656.2000.00448.x

[jane70030-bib-0081] Osojnik, N. , Žagar, A. , Carretero, M. A. , García‐Muñoz, E. , & Vrezec, A. (2013). Ecophysiological dissimilarities of two sympatric lizards. Herpetologica, 69(4), 445–454. 10.1655/HERPETOLOGICA-D-13-00014

[jane70030-bib-0082] Pearson, O. P. , & Bradford, D. F. (1976). Thermoregulation of lizards and toads at high altitudes in Peru. Copeia, 1976(1), 155–170. 10.2307/1443786

[jane70030-bib-0083] Perry, C. , Sarraude, T. , Billet, M. , Minot, E. , Gangloff, E. J. , & Aubret, F. (2024). Sex‐dependent shifts in body size and condition along replicated elevational gradients in a montane colonising ectotherm, the common wall lizard (*Podarcis muralis*). Oecologia, 206, 335–346. 10.1007/s00442-024-05634-8 39523232

[jane70030-bib-0084] Pianka, E. R. (1973). The structure of lizard communities. Annual Review of Ecology and Systematics, 4, 53–74. 10.1146/annurev.es.04.110173.000413

[jane70030-bib-0085] Pianka, E. R. (1981). Competition and niche theory. In R. May & A. Mclean (Eds.), Theoretical ecology: Principles and applications (2nd ed., pp. 167–196). Oxford Press.

[jane70030-bib-0086] Plasman, M. , Bautista, A. , McCue, M. D. , & de la Díaz Vega‐Pérez, A. H. (2020). Resting metabolic rates increase with elevation in a mountain‐dwelling lizard. Integrative Zoology, 15(5), 363–374. 10.1111/1749-4877.12434 32306560

[jane70030-bib-0087] Pollock, L. J. , Tingley, R. , Morris, W. K. , Golding, N. , O'Hara, R. B. , Parris, K. M. , Vesk, P. A. , & McCarthy, M. A. (2014). Understanding co‐occurrence by modelling species simultaneously with a Joint Species Distribution Model (JSDM). Methods in Ecology and Evolution, 5(5), 397–406. 10.1111/2041-210X.12180

[jane70030-bib-0088] Rassati, G. (2010). Contributo alla conoscenza della distribuzione della Lucertola di Horvath Iberolacerta horvathi e della Lucertola dei muri Podarcis muralis in Friuli Venezia Giulia e in Veneto. Atti del museo civico di storia naturale di Trieste, 54(2009), 133–146.

[jane70030-bib-1010] R Core Team . (2024). R: A language and environment for statistical computing. R Foundation for Statistical Computing. https://www.R‐project.org/

[jane70030-bib-0089] Reif, J. , Reifová, R. , Skoracka, A. , & Kuczyński, L. (2018). Competition‐driven niche segregation on a landscape scale: Evidence for escaping from syntopy towards allotopy in two coexisting sibling passerine species. Journal of Animal Ecology, 87(3), 774–789. 10.1111/1365-2656.12808 29430650

[jane70030-bib-0090] Sacchi, R. , Pellitteri‐Rosa, D. , Capelli, A. , Ghitti, M. , Di Paoli, A. , Bellati, A. , Galeotti, P. , Pupin, F. , & Fasola, M. (2012). Studying the reproductive biology of the common wall lizard using ultrasonography. Journal of Zoology, 287(4), 301–310. 10.1111/j.1469-7998.2012.00917.x

[jane70030-bib-0091] Santillán, V. , Quitián, M. , Tinoco, B. A. , Zárate, E. , Schleuning, M. , Böhning‐Gaese, K. , & Neuschulz, E. L. (2018). Spatio‐temporal variation in bird assemblages is associated with fluctuations in temperature and precipitation along a tropical elevational gradient. PLoS One, 13(5), e0196179. 10.1371/journal.pone.0196179 29746478 PMC5945003

[jane70030-bib-1003] Schwarzkopf, L. , Caley, M. J. , & Kearney, M. R. (2016). One lump or two? Explaining a major latitudinal transition in reproductive allocation in a viviparous lizard. Functional Ecology, 30(8), 1373–1383. 10.1111/1365-2435.12622

[jane70030-bib-0092] Sears, M. W. , & Angilletta, M. J. (2004). Body size clines in Sceloporus lizards: Proximate mechanisms and demographic constraints. Integrative and Comparative Biology, 44, 433–442. 10.1093/icb/44.6.433 21676729

[jane70030-bib-0093] Senior, A. F. , Atkins, Z. S. , Clemann, N. , Gardner, M. G. , Schroder, M. , While, G. M. , Wong, B. M. , & Chapple, D. G. (2019). Variation in thermal biology of three closely related lizard species along an elevation gradient. Biological Journal of the Linnean Society, 127(2), 278–291. 10.1093/biolinnean/blz046

[jane70030-bib-0094] Shine, R. (2005). Life‐history evolution in reptiles. Annual Review of Ecology, Evolution, and Systematics, 36, 23–46. https://www.jstor.org/stable/30033795

[jane70030-bib-0095] Sillero, N. , Campos, J. , Bonardi, A. , Corti, C. , Creemers, R. , Crochet, P. , Crnobrnja Isailović, J. , Denoël, M. , Ficetola, G. F. , Gonçalves, J. , Kuzmin, S. , Lymberakis, P. , de Pous, P. , Rodríguez, A. , Sindaco, R. , Speybroeck, J. , Toxopeus, B. , Vieites, D. R. , & Vences, M. (2014). Updated distribution and biogeography of amphibians and reptiles of Europe. Amphibia‐Reptilia, 35(1), 1–31. 10.1163/15685381-00002935

[jane70030-bib-0096] Sindaco, R. , Doria, G. , Razzetti, E. , & Bernini, F. (2006). Atlante degli Anfibi e dei Rettili d'Italia/atlas of Italian amphibians and reptiles. Societas Herpetologica Italica.

[jane70030-bib-0097] Smith, G. R. , & Ballinger, R. E. (1994). Temperature relationships in the high‐altitude viviparous lizard, Sceloporus jarrovi. American Midland Naturalist, 131(1), 181–189. 10.2307/2426621

[jane70030-bib-0098] Somero, G. N. , Lockwood, B. N. , & Tomanek, L. (2017). Biochemical adaptation: Response to environmental challenges from life's origins to the Anthropocene. Sinauer Associates, Incorporated Publishers.

[jane70030-bib-0099] Speybroeck, J. , Beukema, W. , Bok, B. , & Van Der Voort, J. (2016). Field guide to the amphibians and reptiles of Britain and Europe. Bloomsbury Publishing.

[jane70030-bib-0100] Sunday, J. M. , Bates, A. E. , Kearney, M. R. , Colwell, R. K. , Dulvy, N. K. , Longino, J. T. , & Huey, R. B. (2014). Thermal‐safety margins and the necessity of thermoregulatory behavior across latitude and elevation. Proceedings of the National Academy of Sciences of the United States of America, 111(15), 5610–5615. 10.1073/pnas.1316145111 24616528 PMC3992687

[jane70030-bib-0101] Tang, J. , & Zhou, S. (2011). The importance of niche differentiation for coexistence on large scales. Journal of Theoretical Biology, 273(1), 32–36. 10.1016/j.jtbi.2010.12.025 21182846

[jane70030-bib-1015] Tannerfeldt, M. , Elmhagen, B. , & Angerbjörn, A. (2002). Exclusion by interference competition? The relationship between red and arctic foxes. Oecologia, 132(2), 213–220. 10.1007/s00442-002-0967-8 28547354

[jane70030-bib-1005] The MathWorks Inc . (2022). Optimization Toolbox version: 9.4 (R2022b). The MathWorks Inc. https://www.mathworks.com

[jane70030-bib-0102] Tieleman, B. I. , Williams, J. B. , & Bloomer, P. (2003). Adaptation of metabolism and evaporative water loss along an aridity gradient. Proceedings of the Royal Society of London. Series B: Biological Sciences, 270(1511), 207–214. 10.1098/rspb.2002.2205 PMC1691220 12590762

[jane70030-bib-0103] Tokeshi, M. (2009). Species coexistence: Ecological and evolutionary perspectives. John Wiley & Sons.

[jane70030-bib-0104] van der Meer, J. (2006). An introduction to dynamic energy budget (DEB) models with special emphasis on parameter estimation. Journal of Sea Research, 56(2), 85–102. 10.1016/j.seares.2006.03.001

[jane70030-bib-0105] Vrezec, A. , & Tome, D. (2004). Altitudinal segregation between Ural Owl *Strix uralensis* and Tawny Owl *S. aluco*: Evidence for competitive exclusion in raptorial birds. Bird Study, 51(3), 264–269. 10.1080/00063650409461362

[jane70030-bib-0106] Watkins, J. E., Jr. , Cardelús, C. , Colwell, R. K. , & Moran, R. C. (2006). Species richness and distribution of ferns along an elevational gradient in Costa Rica. American Journal of Botany, 93(1), 73–83. 10.3732/ajb.93.1.73

[jane70030-bib-0107] Wickham, H. , Averick, M. , Bryan, J. , Chang, W. , McGowan, L. D. , François, R. , Grolemund, G. , Hayes, A. , Henry, L. , Hester, J. , Kuhn, M. , Pedersen, T. L. , Miller, E. , Bache, S. M. , Müller, K. , Ooms, J. , Robinson, D. , Seidel, D. P. , Spinu, V. , … Yutani, H. (2019). Welcome to the tidyverse. Journal of Open Source Software, 4(43), 1686. 10.21105/joss.01686

[jane70030-bib-0108] Wiens, J. J. , & Donoghue, M. J. (2004). Historical biogeography, ecology and species richness. Trends in Ecology & Evolution, 19(12), 639–644. 10.1016/j.tree.2004.09.011 16701326

[jane70030-bib-1012] Vitt, L. J. , & Caldwell, J. P. (2013). Herpetology: An introductory biology of amphibians and reptiles (4th ed., p. 776). Academic Press.

[jane70030-bib-0109] Žagar, A. (2008). The lowest altitudinal record of Horvath's Rock Lizard (*Iberolacerta horvathi*) in Slovenia. Natura Sloveniae, 10(2), 59–61. 10.14720/ns.10.2.59-61

[jane70030-bib-0110] Žagar, A. (2016). Altitudinal distribution and habitat use of the common wall lizard *Podarcis muralis* (Linnaeus, 1768) and the Horvath's rock lizard *Iberolacerta horvathi* (Méhely, 1904) in the Kočevsko region (S Slovenia). Natura Sloveniae, 18(2), 47–62.

[jane70030-bib-0111] Žagar, A. , Bitenc, K. , Vrezec, A. , & Carretero, M. A. (2015). Predators as mediators: Differential antipredator behavior in competitive lizard species in a multi‐predator environment. Zoologischer Anzeiger‐A Journal of Comparative Zoology, 259, 31–40. 10.1016/j.jcz.2015.10.002

[jane70030-bib-0112] Žagar, A. , Carretero, M. A. , Osojnik, N. , Sillero, N. , & Vrezec, A. (2015). A place in the sun: Interspecific interference affects thermoregulation in coexisting lizards. Behavioral Ecology and Sociobiology, 69, 1127–1137. 10.1007/s00265-015-1927-8

[jane70030-bib-0113] Žagar, A. , Carretero, M. A. , Vrezec, A. , Drašler, K. , & Kaliontzopoulou, A. (2017). Towards a functional understanding of species coexistence: Ecomorphological variation in relation to whole‐organism performance in two sympatric lizards. Functional Ecology, 31(9), 1780–1791. 10.1111/1365-2435.12878

[jane70030-bib-0114] Žagar, A. , Gomes, V. , & Sillero, N. (2023). Selected microhabitat and surface temperatures of two sympatric lizard species. Acta Oecologica, 118, 103887.

[jane70030-bib-0115] Žagar, A. , Kos, I. , & Vrezec, A. (2013). Habitat segregation patterns of reptiles in Northern Dinaric Mountains (Slovenia). Amphibia‐Reptilia, 34(2), 263–268. 10.1163/15685381-00002889

[jane70030-bib-0116] Žagar, A. , Planinc, G. , & Krofel, M. (2007). Records of Horvath's Rock Lizard (*Iberolacerta horvathi*) from the Notranjsko podolje region (central Slovenia). Natura Sloveniae, 9(2), 43–44. 10.14720/ns.9.2.43-44

[jane70030-bib-0117] Žagar, A. , Simčič, T. , Carretero, M. A. , & Vrezec, A. (2015). The role of metabolism in understanding the altitudinal segregation pattern of two potentially interacting lizards. Comparative Biochemistry and Physiology Part A: Molecular & Integrative Physiology, 179, 1–6. 10.1016/j.cbpa.2014.08.018 25194990

[jane70030-bib-0118] Zamora‐Camacho, F. J. , Reguera, S. , Moreno‐Rueda, G. , & Pleguezuelos, J. M. (2013). Patterns of seasonal activity in a Mediterranean lizard along 2200 m altitudinal gradient. Journal of Thermal Biology, 38, 64–69. 10.1016/j.jtherbio.2012.11.002

