# Supplementary material for: Microclimate variability impacts the coexistence of highland and lowland ectotherms
Source: J Anim Ecol. 2025 Mar 20;94(5):999–1013. doi: 10.1111/1365-2656.70030 (PMC12056348; doi:10.1111/1365-2656.70030)
Supplement: Supplementary file 1 — Figure S1. Schematic representation of the metabolic processes in the standard DEB model. Figure S2. Plots of univariate data used in DEB model estimation. Table S1. Life history data and corresponding model predictions for the two studied lizard species: Horvath's rock lizard (Iberolacerta horvathi) and Common wall lizard (Podarcis muralis). Table S2. Table used to estimate temperatures at start of activity for both species of lizards. Table S3. Core DEB parameters for the two studied species of lizards. Table S4. Individual coefficient results of OLS linear regression models of the effect of elevation and location type on environmental variables. Table S5. Individual coefficient results of linear models of the effect of elevation, species, and location type on life history traits when modelled using spring preferred body temperatures. Table S6. Post hoc comparisons of location types when modelled using spring preferred body temperatures. Table S7. Individual coefficient results of linear models of the effect of elevation, species, and location type on life history traits when modelled using summer preferred body temperatures. Table S8. Post hoc comparisons of location types when modelled using summer preferred body temperatures. [file JANE-94-999-s002.docx]

**SUPPLEMENTARY MATERIAL**

Table S1: Life history data and corresponding model predictions for the two studied lizard species: Horvath’s rock lizard (*Iberolacerta horvathi*) and Common wall lizard (*Podarcis muralis*). “Age at hatching” corresponds to incubation time, “age at puberty” corresponds to time between birth and puberty. Personal unpublished data comes from a constantly expanding database of morphometric field observations for both species obtained in the studied locations and is available upon request. Bolded references present sources for closely related species (*Iberolacerta bonnali*).

| **Species:** |  |  | ***Podarcis muralis* (lowland)** | | | ***Iberolacerta horvathi* (highland)** | | |
| --- | --- | --- | --- | --- | --- | --- | --- | --- |
| **Data:** |  | **Unit** | **Observed** | **Predicted** | **Data source** | **Observed** | **Predicted** | **Data source** |
| Age | Birth | day | 65.90 | 63.90 | Van Damme et al., 1992 | 44.00 | 46.44 | Ljubisavljević et al., 2012 |
|  | Puberty | day | 660 | 651 | Castanet and Roche, 1981 | 1095 | 1034 | Bernini and Doria, 2006 |
| Life span |  | day | 3650 | 3647 | Castanet, 1994 | 3285 | 3290 | Bernini and Doria, 2006 |
| SVL | Hatching | cm | 2.41 | 2.6 | in den Bosch and Bout, 1998 | 2.48 | 2.79 | Ljubisavljević et al., 2012 |
|  | Puberty (females) | cm | 4.42 | 4.511 | Castanet and Roche, 1981 | 4.90 | 4.71 | **Arribas and Galán, 2005** |
|  | Puberty (males) | cm | 4.99 | 4.988 | Castanet and Roche, 1981 | 4.50 | 4.50 | **Arribas and Galán, 2005** |
|  | Ultimate (females) | cm | 6.683 | 6.45 | Personal data | 6.77 | 6.39 | Personal data |
|  | Ultimate (males) | cm | 6.223 | 6.264 | Personal data | 6.17 | 6.16 | Personal data |
| Mass | Egg | g | 0.98 | 0.9368 | in den Bosch and Bout, 1998 | 1.30 | 1.26 | in den Bosch and Bout, 1998 |
|  | Hatchling | g | 0.36 | 0.3442 | in den Bosch and Bout, 1998 | 0.46 | 0.40 | in den Bosch and Bout, 1998 |
|  | Ultimate (females) | g | 5.078 | 5.256 | Personal data | 4.64 | 4.84 | Personal data |
|  | Ultimate (males) | g | 5.95 | 5.954 | Personal data | 4.96 | 4.97 | Personal data |
| Maximum reproduction rate |  | egg/day | 0.027 | 0.023 | Ji and Braña, 2000 | 0.01 | 0.01 | Speybroeck et al., 2016 |

Table S2: Table used to estimate temperatures at start of activity for both species of lizards. Temperatures were recorded using a digital thermometer (Fluke^®^ 971, accuracy ±0.5°C). Tair – Air temperature, Tsurf – temperature of the surface in front of the refuge, Tref - temperature within the refuge. F- female, M – male, SAD – subadult, JUV – juvenile. AD -Adult, IM – immature.

| **ID** | **SPECIES** | **SEX** | **AGE** | **T air** | **T surf** | **T ref** |
| --- | --- | --- | --- | --- | --- | --- |
| 1 | IHOR | AD | AD | 24.1 | 34.3 | 19.1 |
| 2 | IHOR | M | AD | 24.0 | 33.3 | 9.7 |
| 3 | IHOR | AD | AD | 24.3 | 23.2 | 19.4 |
| 4 | IHOR | F | AD | 22.6 | 22.4 | 17.0 |
| 5 | IHOR | M | AD | 24.0 | 15.9 | 5.6 |
| 6 | IHOR | SAD | IM | 24.0 | 16.5 | 1.5 |
| 7 | IHOR | AD | AD | 28.0 | 24.3 | 4.5 |
| 8 | PMUR | JUV | IM | 27.4 | 35.8 | 13.1 |
| 9 | PMUR | F | AD | 21.0 | 38.0 | 15.0 |
| 12 | IHOR | M | AD | 23.7 | 35.1 | 3.8 |
| 13 | PMUR | F | AD | 25.5 | 11.1 | 2.2 |
| 17 | IHOR | M | AD | 17.0 | 18.9 | 7.8 |
| 18 | IHOR | M | AD | 18.8 | 24.7 | 11.4 |
| 19 | IHOR | F | AD | 23.3 | 22.8 | 7.0 |
| 20 | IHOR | M | AD | 23.3 | 35.4 | 6.8 |
| 21 | PMUR | F | AD | 23.2 | 19.9 | 13.3 |
| 22 | IHOR | M | AD | 23.2 | 24.9 | 6.1 |
| 24 | IHOR | F | AD | 26.7 | 20.0 | 2.0 |
| 25 | IHOR | SAD | IM | 26.6 | 31.7 | 16.1 |
| 26 | PMUR | F | AD | 26.8 | 24.0 | 18.0 |
| 27 | PMUR | M | AD | 20.4 | 29.5 | 15.1 |
| 28 | PMUR | M | AD | 21.1 | 30.4 | 15.6 |
| 29 | PMUR | M | AD | 21.1 | 21.1 | 17.0 |
| 30 | PMUR | F | AD | 21.7 | 27.3 | 15.9 |
| 31 | IHOR | JUV | IM | 24.0 | 20.2 | 17.6 |
| 32 | IHOR | JUV | IM | 26.1 | 20.1 | 6.2 |
| 33 | IHOR | FGR | AD | 27.0 | 37.0 | 8.0 |
| 34 | PMUR | F | AD | 24.3 | 36.7 | 21.0 |
| 35 | PMUR | F | AD | 24.3 | 22.8 | 21.6 |
| 36 | IHOR | M | AD | 25.2 | 34.2 | 20.6 |
| 38 | IHOR | F | AD | 19.5 | 17.6 | 14.2 |
| 39 | IHOR | FGR | AD | 20.1 | 38.5 | 22.6 |
| 40 | IHOR | F | AD | 20.3 | 35.4 | 20.3 |
| 41 | IHOR | M | AD | 20.4 | 30.0 | 18.1 |
| 42 | IHOR | SAD | IM | 21.3 | 38.4 | 18.3 |
| 43 | IHOR | M | AD | 21.2 | 29.8 | 21.2 |
| 44 | IHOR | FGR | AD | 24.5 | 25.3 | 21.0 |
| 45 | IHOR | FGR | AD | 24.5 | 41.0 | 14.0 |
| 46 | IHOR | FGR | AD | 25.4 | 21.6 | 13.3 |
| 47 | IHOR | FGR | AD | 24.6 | 27.9 | 16.3 |
| 48 | IHOR | FGR | AD | 25.0 | 22.1 | 14.0 |
| 49 | IHOR | JUV | IM | 25.1 | 38.3 | 26.0 |
| 50 | IHOR | M | AD | 28.0 | 34.0 | 23.0 |
| 51 | IHOR | M | AD | 28.0 | 42.0 | 20.0 |
| 52 | IHOR | JUV | IM | 28.0 | 27.2 | 17.0 |
| 53 | IHOR | F | AD | 30.1 | 29.0 | 19.0 |
| 55 | IHOR | JUV | IM | 25.4 | 28.9 | 16.3 |
| 56 | IHOR | JUV | IM | 26.0 | 31.7 | 21.0 |
| 57 | PMUR | F | AD | 31.6 | 37.7 | 30.0 |
| 58 | IHOR | F | AD | 29.3 | 35.5 | 11.4 |
| 59 | IHOR | M | AD | 30.6 | 27.6 | 20.3 |
| 60 | IHOR | M | AD | 30.7 | 42.9 | 30.2 |
| 61 | IHOR | M | AD | 32.0 | 24.8 | 18.1 |
| 62 | PMUR | M | AD | 32.0 | 42.2 | 32.7 |
| 64 | IHOR | M | AD | 32.0 | 26.5 | 21.1 |
| 65 | IHOR | JUV | IM | 29.6 | 55.0 | 24.1 |
| 66 | IHOR | M | AD | 29.6 | 34.0 | 16.7 |
| 68 | IHOR | M | AD | 23.2 | 32.3 | 23.6 |
| 69 | IHOR | M | AD | 23.9 | 29.8 | 25.4 |
| 70 | PMUR | M | AD | 22.2 | 40.9 | 25.7 |
| 71 | PMUR | F | AD | 29.2 | 25.0 | 21.3 |
| 72 | IHOR | JUV | IM | 29.2 | 31.7 | 14.9 |
| 73 | IHOR | JUV | IM | 25.8 | 24.6 | 18.2 |
| 74 | IHOR | SAD | IM | 27.0 | 26.2 | 20.7 |
| 75 | IHOR | SAD | IM | 29.4 | 27.5 | 16.0 |
| 77 | IHOR | M | AD | 29.4 | 29.3 | 22.8 |
| 79 | IHOR | SAD | IM | 29.4 | 20.6 | 11.6 |
| 81 | IHOR | F | AD | 30.0 | 28.2 | 8.6 |
| 82 | IHOR | F | AD | 30.0 | 50.0 | 6.5 |
| 83 | IHOR | SAD | IM | 31.4 | 25.2 | 18.0 |
| 84 | IHOR | JUV | IM | 31.4 | 22.5 | 6.8 |
| 85 | IHOR | M | AD | 25.3 | 30.4 | 24.0 |
| 88 | IHOR | SAD | IM | 29.3 | 22.1 | 16.4 |
| 89 | IHOR | F | AD | 27.2 | 21.2 | 18.3 |
| 90 | IHOR | M | AD | 30.3 | 22.8 | 19.4 |
| 92 | IHOR | M | AD | 30.3 | 31.1 | 18.0 |
| 93 | IHOR | M | AD | 31.0 | 21.6 | 10.6 |
| 94 | IHOR | M | AD | 31.0 | 20.1 | 11.0 |
| 95 | IHOR | SAD | IM | 31.0 | 34.3 | 19.2 |
| 96 | IHOR | SAD | IM | 31.0 | 44.4 | 10.9 |
| 97 | IHOR | F | AD | 30.4 | 30.8 | 21.5 |
| 98 | IHOR | M | AD | 30.4 | 23.6 | 11.5 |
| 99 | IHOR | SAD | IM | 30.4 | 25.7 | 10.8 |
| 100 | PMUR | F | AD | 30.7 | 24.1 | 19.9 |
| 101 | IHOR | M | AD | 30.7 | 22.9 | 17.6 |
| 102 | IHOR | M | AD | 30.7 | 25.7 | 29.8 |
| 103 | PMUR | F | AD | 28.0 | 26.8 | 25.7 |
| 104 | IHOR | M | AD | 33.3 | 34.3 | 24.3 |
| 105 | PMUR | F | AD | 30.0 | 36.9 | 29.2 |
| 106 | IHOR | SAD | IM | 29.0 | 36.3 | 32.0 |
| 108 | IHOR | SAD | IM | 32.0 | 30.7 | 34.3 |
| 109 | IHOR | M | AD | 25.0 | 32.0 | 16.0 |
| 110 | IHOR | AD | AD | 28.0 | 24.0 | 15.0 |
| 111 | IHOR | F | AD | 28.0 | 24.0 | 19.0 |
| 112 | IHOR | JUV | IM | 28.0 | 48.0 | 11.0 |
| 113 | PMUR | F | AD | 28.0 | 30.0 | 17.0 |
| 114 | IHOR | SAD | IM | 27.0 | 22.0 | 15.0 |
| 115 | IHOR | M | AD | 28.0 | 26.0 | 19.0 |
| 116 | IHOR | JUV | IM | 28.0 | 44.0 | 12.0 |
| 117 | IHOR | M | AD | 27.0 | 26.0 | 13.0 |
| 119 | IHOR | M | AD | 27.0 | 18.0 | 19.0 |
| 120 | IHOR | SAD | IM | 27.0 | 22.0 | 24.0 |
| 121 | IHOR | F | AD | 27.0 | 23.0 | 18.0 |
| 122 | IHOR | SAD | IM | 24.0 | 22.0 | 19.0 |
| 123 | IHOR | M | AD | 24.0 | 28.0 | 15.0 |
| 125 | IHOR | M | AD | 26.0 | 22.0 | 14.0 |
| 126 | IHOR | F | AD | 27.0 | 35.0 | 12.0 |
| 127 | IHOR | AD | AD | 28.0 | 41.0 | 16.0 |
| 130 | IHOR | M | AD | 28.0 | 18.0 | 11.0 |
| 134 | IHOR | SAD | IM | 28.0 | 31.0 | 22.0 |
| 137 | PMUR | JUV | IM | 20.0 | 13.0 | 12.0 |
| 140 | IHOR | SAD | IM | 21.0 | 19.0 | 7.0 |
| 141 | IHOR | M | AD | 20.0 | 31.0 | 16.0 |
| 142 | IHOR | JUV | IM | 21.0 | 39.0 | 18.0 |
| 143 | IHOR | M | AD | 22.0 | 22.0 | 12.0 |
| 144 | IHOR | SAD | IM | 22.0 | 33.0 | 8.0 |
| 145 | IHOR | M | AD | 22.0 | 17.0 | 10.0 |
| 147 | IHOR | M | AD | 23.0 | 24.0 | 14.0 |
| 150 | IHOR | F | AD | 23.0 | 45.0 | 8.0 |
| 151 | IHOR | M | AD | 23.0 | 32.0 | 23.0 |
| 153 | IHOR | F | AD | 24.0 | 27.0 | 12.0 |
| 154 | IHOR | F | AD | 24.0 | 34.0 | 13.0 |
| 160 | IHOR | F | AD | 24.0 | 40.0 | 11.0 |
| 161 | IHOR | AD | AD | 24.0 | 19.0 | 12.0 |
| 165 | PMUR | SAD | IM | 23.0 | 27.0 | 18.0 |
| 166 | PMUR | M | AD | 24.0 | 17.0 | 20.0 |

Table S3: Core DEB parameters for the two studied species of lizards. Parameter estimation was carried out using the covariation method (Lika et al., 2011; Marques et al., 2018).

|  | ***Podarcis muralis*** | ***Iberolacerta horvathi*** |  |
| --- | --- | --- | --- |
| **Parameter** | **Estimate** | | **Units** |
| z, zoom factor (relative volumetric length) | 1.3094 | 1.4959 | - |
| δ_M_, shape correction factor | 0.20302 | 0.23419 | - |
| v, energy conductance | 0.027014 | 0.048738 | cm day^-1^ |
| κ, allocation fraction to soma | 0.2756 | 0.31132 | - |
| [ṗ_M_], somatic maintenance | 52.6527 | 31.1886 | J cm^-3^day^-1^ |
| maturity maintenance rate coefficient | 0.002 | 0.002 | day^-1^ |
| [E_G_], cost of structure | 7835.98 | 7838.15 | J cm^-3^ |
| maturity at birth | 3480 | 5108 | J |
| maturity at puberty | 29030 | 34480 | J |

Table S4: Individual coefficient results of OLS linear regression models of the effect of elevation and location type on environmental variables

|  | **Mean temperature** | | |
| --- | --- | --- | --- |
| *Predictors* | *Estimates* | *SE* | *P* |
| Intercept | 14.1 | 0.581 | <0.01*** |
| Elevation | -0.005 | 0.0005 | <0.01*** |
| Loc. type:Pmur allotopy | -0.250 | 0.384 | 0.528 |
| Loc. type:Sintopy | 0.819 | 0.403 | 0.067˙ |
| F(3, 11): 65.2; R-squared: 0.932; p-value: <0.01 | | | |
|  | **Mean relative humidity** | | |
| *Predictors* | *Estimates* | *SE* | *P* |
| Intercept | 65.0 | 2.92 | <0.01*** |
| Elevation | 0.015 | 0.002 | <0.01*** |
| Loc. type:Pmur allotopy | 3.22 | 1.93 | 0.123 |
| Loc. type:Sintopy | -1.23 | 3.03 | 0.555 |
| F(3, 11): 17.68; R-squared: 0.828; p-value: <0.01 | | | |
|  | **Mean solar radiation** | | |
| *Predictors* | *Estimates* | *SE* | *P* |
| Intercept | 81.3 | 9.50 | <0.01*** |
| Elevation | 0.003 | 0.008 | 0.773 |
| Loc. type:Pmur allotopy | 2.12 | 6.28 | 0.742 |
| Loc. type:Sintopy | -4.36 | 6.0 | 0.523 |
| F(3, 11): 24.191; R-squared: 0.570 ; p-value: 0.646 | | | |
|  | **Days without snow cover** | | |
| *Predictors* | *Estimates* | *SE* | *P* |
| Intercept | 5824 | 119 | <0.01*** |
| Elevation | -1.053 | 0.106 | <0.01*** |
| Loc. type:Pmur allotopy | 48.4 | 78.7 | 0.551 |
| Loc. type:Sintopy | 89.3 | 82.7 | 0.304 |
| F(3, 11):54.342; R-squared:0.937 ; p-value: <0.01 | | | |

Table S5: Individual coefficient results of linear models of the effect of elevation, species, and location type on life history traits when modelled using spring preferred body temperatures.

|  | **Egg development time** | | |
| --- | --- | --- | --- |
| *Predictors* | *Estimates* | *SE* | *p* |
| **Intercept** | **33.9** | **2.06** | **<0.01***** |
| **Elevation** | **0.02** | **0.002** | **<0.01***** |
| **Species:P. muralis** | **8.53** | **0.958** | **<0.01***** |
| **Loc. type:Pmur allotopy** | **2.76** | **1.321** | **0.05*** |
| **Loc. type:Sintopy** | **-3.32** | **1.389** | **0.03*** |
| F(4, 25):84.48; adjusted R-squared:0.92.; p-value: <0.001 | | | |
|  | **Lifespan** | | |
| *Predictors* | *Estimates* | *SE* | *p* |
| **Intercept** | **5.77** | **0.279** | **<0.01***** |
| **Elevation** | **0.002** | **0.0002** | **<0.01***** |
| **Species:P. muralis** | **0.93** | **0.130** | **<0.01***** |
| Loc. type:Pmur allotopy | -0.24 | 0.179 | 0.283 |
| **Loc. type:Sintopy** | **-042** | **0.188** | **0.282*** |
| F(4, 25):37.16; adjusted R-squared:0.83.; p-value: <0.001 | | | |
|  | **Years reproducing** | | |
| *Predictors* | *Estimates* | *SE* | *p* |
| **Intercept** | **2.54** | **0.25** | **<0.001***** |
| Elevation | -0.0003 | 0.0003 | 0.36 |
| **Species:P. muralis** | **1.73** | **0.18** | **0.001***** |
| F(4, 25):47.48; adjusted R-squared: 0.76.; p-value: 0.0058 | | | |
|  | **Yearly basking time** | | |
| *Predictors* | *Estimates* | *SE* | *p* |
| **Intercept** | **1666** | **88.9** | **<0.001***** |
| **Elevation** | **-0.26** | **0.08** | **<0.01***** |
| **Species:P. muralis** | **-348** | **41.3** | **<0.001***** |
| Loc. type:Pmur allotopy | -20.3 | 57.1 | 0.291 |
| **Loc. type:Sintopy** | **143** | **60.1** | **0.025*** |
| F(4, 25):22.29; adjusted R-squared:0.75.; p-value: <0.001 | | | |
|  | **Yearly foraging time** | | |
| *Predictors* | *Estimates* | *SE* | *p* |
| **Intercept** | **459** | **34.1** | **<0.001***** |
| **Elevation** | **-0.12** | **0.03** | **<0.01***** |
| **Species:P. muralis** | **210** | **35.64** | **<0.001***** |
| Loc. type:Pmur allotopy | -9.63 | 29.2 | 0.62 |
| **Loc. type:Sintopy** | **49.9** | **20.2** | **0.02**** |
| Elevation:SpeciesP. muralis | -0.08 | 0.04 | 0.07 |
| F(5, 24):40.1; adjusted R-squared:0.87.; p-value: <0.001 | | | |
|  | **Yearly fecundity** | | |
| *Predictors* | *Estimates* | *SE* | *p* |
| **Intercept** | **0.69** | **0.29** | **<0.05**** |
| Elevation | -0.0003 | 0.0003 | <0.069 |
| **Species:P. muralis** | **2.39** | **0.30** | **<0.001***** |
| Loc. type:Pmur allotopy | -0.22 | 0.16 | 0.069 |
| **Loc. type:Sintopy** | **0.36** | **0.17** | **<0.05**** |
| **Elevation:SpeciesP. muralis** | **-0.001** | **0.0004** | **<0.01***** |
| F(5, 24):46.49; adjusted R-squared:0.88.; p-value: <0.001 | | | |

Table S6: Post hoc comparisons of location types when modelled using spring preferred body temperatures

| ***Post hoc comparison*** | ***Estimates*** | ***SE*** | ***p*** |
| --- | --- | --- | --- |
| **Egg development time** | | | |
| *P. muralis* allotopy - *I. horvathi* allotopy | 2.76 | 1,32 | 0.11 |
| Sintopy - *I. horvathi* allotopy | -3.32 | 1.39 | 0.06 |
| **Sintopy - *P. muralis* allotopy** | **-6.08** | **1.78** | **<0.001***** |
| **Lifespan** | | | |
| *P. muralis* allotopy - *I. horvathi* allotopy | -0.24 | 0.18 | 0.38 |
| Sintopy - *I. horvathi* allotopy | -0.41 | 0.19 | 0.08 |
| Sintopy - *P. muralis* allotopy | -0.18 | 0.16 | 0.52 |
| **Yearly basking time** | | | |
| *P. muralis* allotopy - *I. horvathi* allotopy | -20.3 | 57.1 | 0.93 |
| Sintopy - *I. horvathi* allotopy | 142 | 60.1 | 0.06 |
| **Sintopy - *P. muralis* allotopy** | **163** | **50.9** | **<0.01**** |
| **Yearly foraging time** | | | |
| *P. muralis* allotopy - *I. horvathi* allotopy | -9.63 | 19.2 | 0.87 |
| Sintopy - *I. horvathi* allotopy | 49.9 | 20.2 | 0.05 |
| **Sintopy - *P. muralis* allotopy** | **59.5** | **17.1** | **<0.01**** |
| **Yearly fecundity** | | | |
| *P. muralis* allotopy - *I. horvathi* allotopy | -0.22 | 0.16 | 0.37 |
| Sintopy - *I. horvathi* allotopy | 0.36 | 0.17 | 0.10 |
| **Sintopy - *P. muralis* allotopy** | **0.58** | **0.14** | **<0.01**** |

Table S7: Individual coefficient results of linear models of the effect of elevation, species, and location type on life history traits when modelled using summer preferred body temperatures.

|  | **Egg development time** | | |
| --- | --- | --- | --- |
| *Predictors* | *Estimates* | *SE* | *p* |
| **Intercept** | **33.9** | **2.06** | **<0.01***** |
| **Elevation** | **0.02** | **0.002** | **<0.01***** |
| **Species:P. muralis** | **8.53** | **0.958** | **<0.01***** |
| **Loc. type:Pmur allotopy** | **2.76** | **1.321** | **0.05*** |
| **Loc. type:Sintopy** | **-3.32** | **1.389** | **0.03*** |
| F(4, 25):84.48; adjusted R-squared:0.92.; p-value: <0.01 | | | |
|  | **Lifespan** | | |
| *Predictors* | *Estimates* | *SE* | *p* |
| **Intercept** | **5.31** | **0.284** | **<0.01***** |
| **Elevation** | **0.001** | **0.0002** | **<0.01***** |
| **Species:P. muralis** | **0.600** | **0.132** | **<0.01***** |
| Loc. type:Pmur allotopy | 0.200 | 0.182 | 0.283 |
| Loc. type:Sintopy | -0.211 | 0.192 | 0.282 |
| F(4, 25):15.66; adjusted R-squared:0.83.; p-value: <0.01 | | | |
|  | **Years reproducing** | | |
| *Predictors* | *Estimates* | *SE* | *p* |
| **Intercept** | **3.153** | **0.350** | **<0.01***** |
| **Elevation** | **-0.001** | **0.0004** | **0.01**** |
| Species:P. muralis | 0.400 | 0.30 | 0.128 |
| F(4, 25):6.261; adjusted R-squared: 0.266.; p-value: 0.0058 | | | |
|  | **Yearly basking time** | | |
| *Predictors* | *Estimates* | *SE* | *p* |
| **Intercept** | **1830** | **104** | **<0.01***** |
| **Elevation** | **-0.35** | **0.09** | **<0.01***** |
| **Species:P. muralis** | **-365** | **48.54** | **<0.01***** |
| Loc. type:Pmur allotopy | -72.4 | 67.1 | 0.291 |
| Loc. type:Sintopy | 92.9 | 70.57 | 0.200 |
| F(4, 25):22.29; adjusted R-squared:0.75.; p-value: <0.01 | | | |
|  | **Yearly foraging time** | | |
| *Predictors* | *Estimates* | *SE* | *p* |
| **Intercept** | **506** | **34.8** | **<0.01***** |
| **Elevation** | **-0.16** | **0.03** | **<0.01***** |
| **Species:P. muralis** | **63.8** | **16.2** | **<0.01***** |
| Loc. type:Pmur allotopy | -27.6 | 22.4 | 0.23 |
| Loc. type:Sintopy | 36.7 | 23.5 | 0.13 |
| F(4, 25):19.27; adjusted R-squared:0.72.; p-value: <0.01 | | | |
|  | **Yearly fecundity** | | |
| *Predictors* | *Estimates* | *SE* | *p* |
| **Intercept** | **0.87** | **0.24** | **<0.01***** |
| Elevation | -0.0004 | 0.0002 | <0.069 |
| **Species:P. muralis** | **1.14** | **0.25** | **<0.01***** |
| Loc. type:Pmur allotopy | -0.26 | 0.13 | 0.069 |
| Loc. type:Sintopy | 0.28 | 0.14 | 0.053 |
| **Elevation:SpeciesP. muralis** | **-0.0009** | **0.0003** | **<0.01***** |
| F(5, 24):18.69; adjusted R-squared:0.75.; p-value: <0.01 | | | |

Table S8: Post hoc comparisons of location types when modelled using summer preferred body temperatures

| ***Post hoc comparison*** | ***Estimates*** | ***SE*** | ***p*** |
| --- | --- | --- | --- |
| **Egg development time** | | | |
| *P. muralis* allotopy - *I. horvathi* allotopy | 2.76 | 1,32 | 0.11 |
| Sintopy - *I. horvathi* allotopy | -3.32 | 1.39 | 0.06 |
| **Sintopy - *P. muralis* allotopy** | **-6.08** | **1.78** | **<0.001***** |
| **Lifespan** | | | |
| *P. muralis* allotopy - *I. horvathi* allotopy | -0.20 | 0.18 | 0.52 |
| Sintopy - *I. horvathi* allotopy | -0.21 | 0.19 | 0.52 |
| **Sintopy - *P. muralis* allotopy** | **-0.41** | **0.16** | **<0.05*** |
| **Yearly basking time** | | | |
| *P. muralis* allotopy - *I. horvathi* allotopy | -72.4 | 67.2 | 0.53 |
| Sintopy - *I. horvathi* allotopy | 92.8 | 70.6 | 0.39 |
| **Sintopy - *P. muralis* allotopy** | **165.2** | **59.9** | **<0.05*** |
| **Yearly foraging time** | | | |
| *P. muralis* allotopy - *I. horvathi* allotopy | -27.62 | 22.40 | 0.44 |
| Sintopy - *I. horvathi* allotopy | 36.68 | 23.55 | 0.28 |
| **Sintopy - *P. muralis* allotopy** | **64.29** | **19.97** | **<0.01**** |
| **Yearly fecundity** | | | |
| *P. muralis* allotopy - *I. horvathi* allotopy | -0.25 | 0.13 | 0.16 |
| Sintopy - *I. horvathi* allotopy | 0.28 | 0.14 | 0.13 |
| **Sintopy - *P. muralis* allotopy** | **0.54** | **0.12** | **<0.001***** |


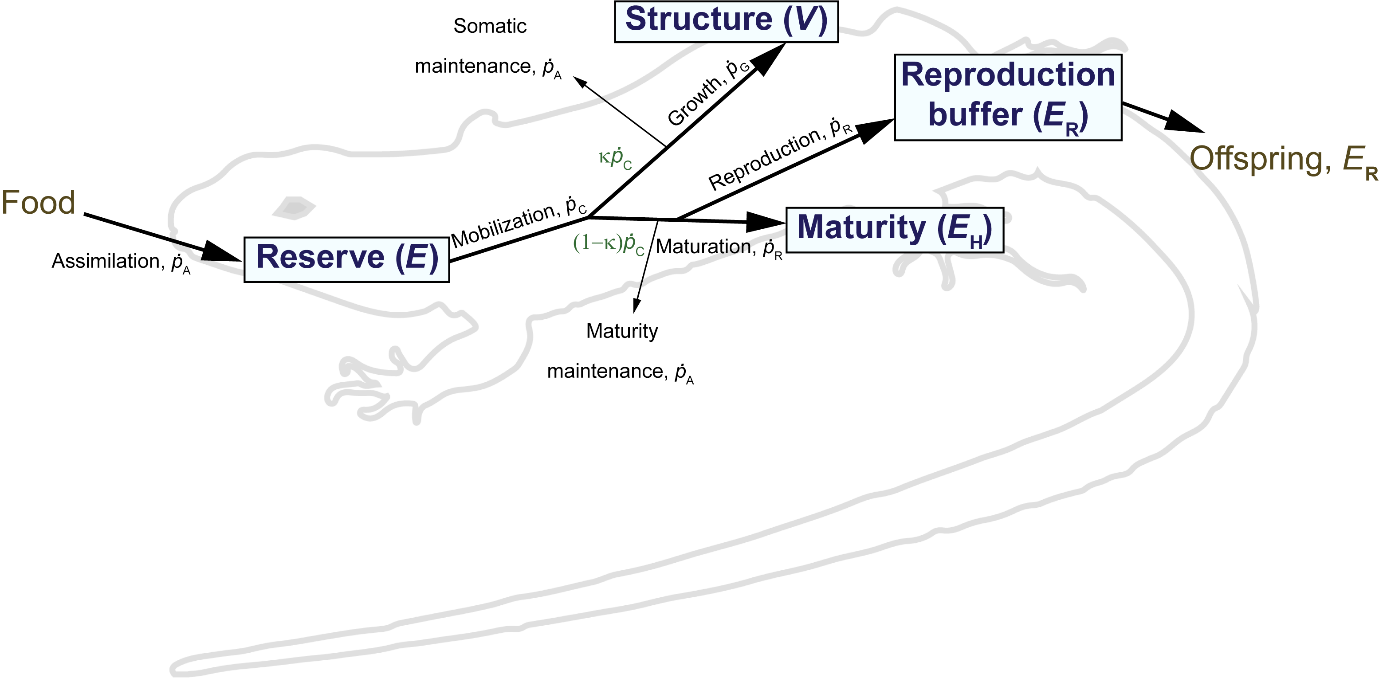


Figure S1: Schematic representation of the metabolic processes in the standard DEB model. Solid arrows represent energy fluxes, boxes mark state variables and green text describes the κ rule. Energy contained in food is assimilated into reserve and further allocated to fueling the metabolic processes. A fixed fraction κ is used for somatic maintenance and growth, the rest ((1-κ) is used for maturation and after puberty for reproduction. standard DEB model of metabolism, showing processes (black text), state variables (boxes) and energy fluxes $\dot{p}$. Modified from Kearney et al, 2013.


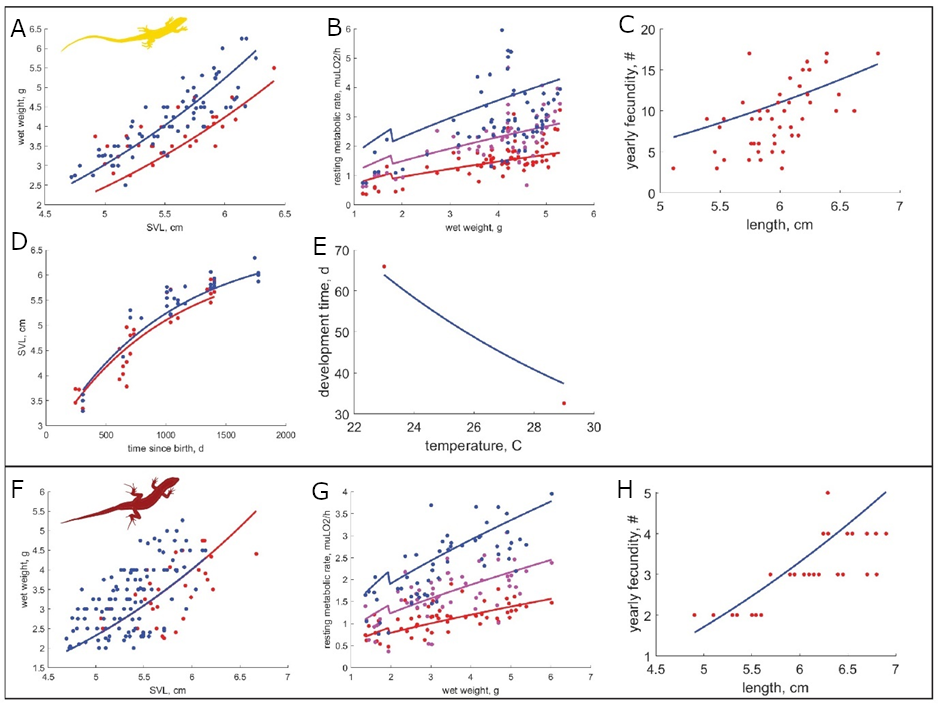


Figure S2: Plots of univariate data used in DEB model estimation. Dots are observations sourced from literature or unpublished data and lines are model predictions. Plots A-E correspond to *Podarcis muralis* and plots F-H to *Iberolacerta horvathi*. In plots A, D and F, red denotes females and blue males. In plots B and G colors correspond to three different temperature regimes; red - 23°C, magenta – 28°C and blue - 33°C. SVL is an abbreviation for snout vent length, yearly fecundity is expressed in number of eggs per year. The overall goodness of fit (SMSE) had a value of 0.010 for *P. muralis* and 0.013 for *I. horvathi*. Both species share a low value of κ; 0.311 for *I. horvathi* and 0.276 for *P. muralis*, pointing to a trend of higher energy investment into maturation and reproduction over maintenance and growth. Maturity levels at birth and puberty were slightly higher in *I. horvathi*, which also exhibits a lower cost of somatic maintenance.

SUPPLEMENTARY REFERENCES

Arribas, O., & Galán, P. (2005). Reproductive characteristics of the Pyrenean high-mountain lizards: Iberolacerta aranica (Arribas, 1993), I. aurelioi (Arribas, 1994) and I. bonnali (Lantz, 1927). Animal Biology, 55(2), 163-190. <https://doi.org/10.1163/1570756053993505>

Bernini, F., Doria, G., Razzetti, E., & Sindaco, R. (2006). Atlante degli Anfibi e dei Rettili d'Italia. Atlas of Italian Amphibians and.

Castanet, J. & Roche, E. (1981) Détermination de l’âge chez le lézard des murailles, Lacerta muralis (Laurenti, 1768) au moyen de la squelettochronologie. Rev. Suisse Zool., 88, 215-226

Castanet, J. (1994) Age estimation and longevity in reptiles. Gerontology, 40, 174-192.

Herman, A. J., & Bout, R. G. (1998). Relationships between maternal size, egg size, clutch size, and hatchling size in European lacertid lizards. Journal of Herpetology, 410-417.

Lika, K., Kearney, M. R., Freitas, V., van der Veer, H. W., van der Meer, J., Wijsman, J. W., Pecquerie, L., & Kooijman, S. A. (2011). The “covariation method” for estimating the parameters of the standard Dynamic Energy Budget model I: Philosophy and approach. Journal of Sea Research, 66(4), 270-277. <https://doi.org/10.1016/J.SEARES.2011.07.010>

Ljubisavljevic., Glasovi, PO., Kalan, K., and Krystufek, B. (2012). Female reproductive characteristics of the Horvathss rock lizard emph(iberolacerta horvathi) from Slovenia. Archives of Biological Sciences. <https://doi.org/10.2298/ABS1202639L>

Marques, G. M., Augustine, S., Lika, K., Pecquerie, L., Domingos, T., & Kooijman, S. A. (2018). The AmP project: comparing species on the basis of dynamic energy budget parameters. PLoS computational biology, 14(5), e1006100. <https://doi.org/10.1371/journal.pcbi.1006100>

Van Damme, R., BAUwENS, D. I. R. K., Braña, F., & Verheyen, R. F. (1992). Incubation temperature differentially affects hatching time, egg survival, and hatchling performance in the lizard Podarcis muralis. Herpetologica, 220-228.
